# Enhanced 2D MoTe_2_ Analogue Switching through Laser Processing and ALD-Passivation for Dual-Function Neuromorphic Devices

**DOI:** 10.1021/acs.nanolett.5c05915

**Published:** 2025-12-15

**Authors:** Mohamed Radwan, Seyed Hossein Hosseini-Shokouh, Abde Mayeen Shafi, Catarina Dias, Fooqia Khalid, Shreya Rajeevan, Henri Hyttinen, Fida Ali, Faisal Ahmed, Zhipei Sun, Harri Lipsanen

**Affiliations:** † Department of Electronics and Nanoengineering 174277 Aalto University, Tietotie 3 FI-02150, Finland; ‡ IFIMUP, Departamento de Física e Astronomia, Faculdade de Ciências, Universidade do Porto, Rua do Campo Alegre s/n, 4169-007 Porto, Portugal; § QTF Centre of Excellence, Department of Applied Physics 174277 Aalto University, Aalto FI-00076, Finland

**Keywords:** Memristor, Memtransistor, Neuromorphic, 2D Materials, Analogue Switching, Laser Patterning, Atomic Layer Deposition

## Abstract

Neuro-based computing, enabled by memristors and memtransistors, has drawn significant attention, with two-dimensional (2D) molybdenum ditelluride (MoTe_2_) emerging as a promising candidate for its low phase-change energy barrier and tunable electrical behavior. However, realizing the full potential of MoTe_2_ for neuromorphic applications has been hindered by the lack of a sufficiently large memory window in pristine devices. In this work, we present an effective method to significantly enhance the analogue switching of lateral MoTe_2_ devices. This method involves two sequential processes: laser treatment followed by the atomic layer deposition of Al_2_O_3_. The synergistic effects of both processes yielded significant performance improvements, with a 70-fold improvement in dynamic range when operating as a memristor and a 20-fold enhancement when functioning as a memtransistor. Consequently, artificial neural network simulations demonstrated a 10-fold enhancement in pattern recognition accuracy, while the dual memristor/memtransistor capability enabled the emulation of both homosynaptic and heterosynaptic plasticity.

## Graphical abstract

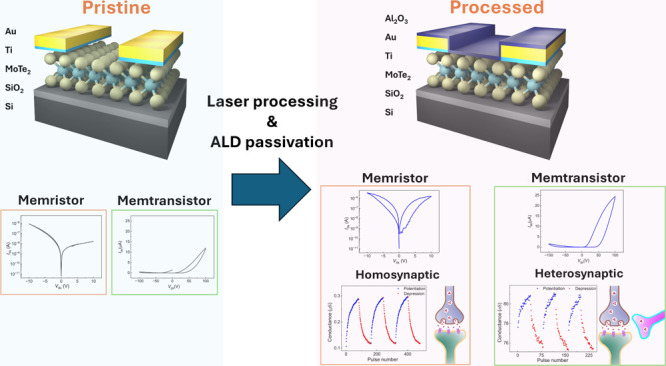

In recent years, the rapid advancement of artificial intelligence (AI), the Internet of Things (IoT), and machine learning has led to a sharp rise in computing demands, significantly increasing energy consumption and the amount of processed data. To address these challenges, neuromorphic computing, utilizing brain-inspired devices, has gained attention as a promising solution. In particular, memristors and memtransistors, which mimic synaptic behavior, exhibit low power consumption, high integration density, and brain-like operational capabilities, making them ideal nanodevices for emerging neuromorphic computing.^1–6^

A wide range of materials have been investigated to build memristors and memtransistors—from traditional oxide materials^7,8^ to new materials such as perovskite, organic, ferroelectric, and two-dimensional (2D) materials.^9–12^ In particular, 2D materials have drawn significant attention for their flexibility, excellent electrical properties, strong gate tunability, and atomically thin nature, making them a compelling choice for neuromorphic devices.^13–15^

Owing to their tunable energy bandgap and significant surface-to-volume ratio, 2D materials offer a high degree of freedom for device optimization through postprocessing methods.^16–21^ Among 2D materials, molybdenum ditelluride (MoTe_2_) offers distinct advantages, including a significantly lower phase change energy barrier,^21,22^ a bandgap closely matching the bandgap of silicon,^23^ and tunable electrical behavior.^24^ However, utilizing MoTe_2_’s full potential for memory applications remains challenging due to the limited memory window and dynamic range (DR) in pristine devices.

Oxidation has been used to generate or enhance the DR characteristics in MoTe_2_ devices by introducing defects into the material.^25^ However, severe oxidation no longer enhances the DR and can degrade device performance. Therefore, a process that can further improve the DR without compromising device performance is highly desirable.

In this study, we employed a two-step treatment method to tailor the properties of mechanically exfoliated MoTe_2_, resulting in a high-performance single device that functions as a memristor and a memtransistor. The material was first treated using a 532 nm continuous wave (CW) laser to enhance current and oxygen absorption and then capped with a 20 nm layer of Al_2_O_3_ deposited via atomic layer deposition (ALD) to introduce acceptor-like trap sites at the Al_2_O_3_/MoTe_2_ interface. Compared with pristine MoTe_2_, the processed device showed significant enhancement in both maximum current and DR behavior. Optimizing the process parameters (e.g., illumination time and power, material thickness, and treatment sequencing) results in considerable improvement: for the memristor, the DR ratio increased by 70-fold and maximum current by 4-fold; for the memtransistor, the DR ratio demonstrated a 20-fold enhancement and maximum current showed a 2.5-fold improvement. Utilized as an artificial synapse, the processed device achieved a 10-fold enhancement in the recognition rate accuracy compared to the pristine device after 45 training epochs. Furthermore, the processed device uniquely combines the functions of both a memristor and a memtransistor in a single unit. This dual functionality allows it to successfully mimic both homosynaptic and heterosynaptic plasticity, mechanisms crucial for normal nervous system operation. Our work demonstrates a practical and feasible method to improve the memristive performance of 2D materials.

Figure 1a shows the schematic structure of the fabricated MoTe_2_ device on a standard Si/SiO_2_ substrate. The pristine MoTe_2_ is subjected to laser treatment before being capped with Al_2_O_3_ via ALD. An optical microscopy image of the main device with a 3 μm channel length is presented in Figure S1. The height profile of the MoTe_2_ flake was measured with an atomic force microscope (AFM), as shown in the inset of Figure S1.

**Figure 1:**
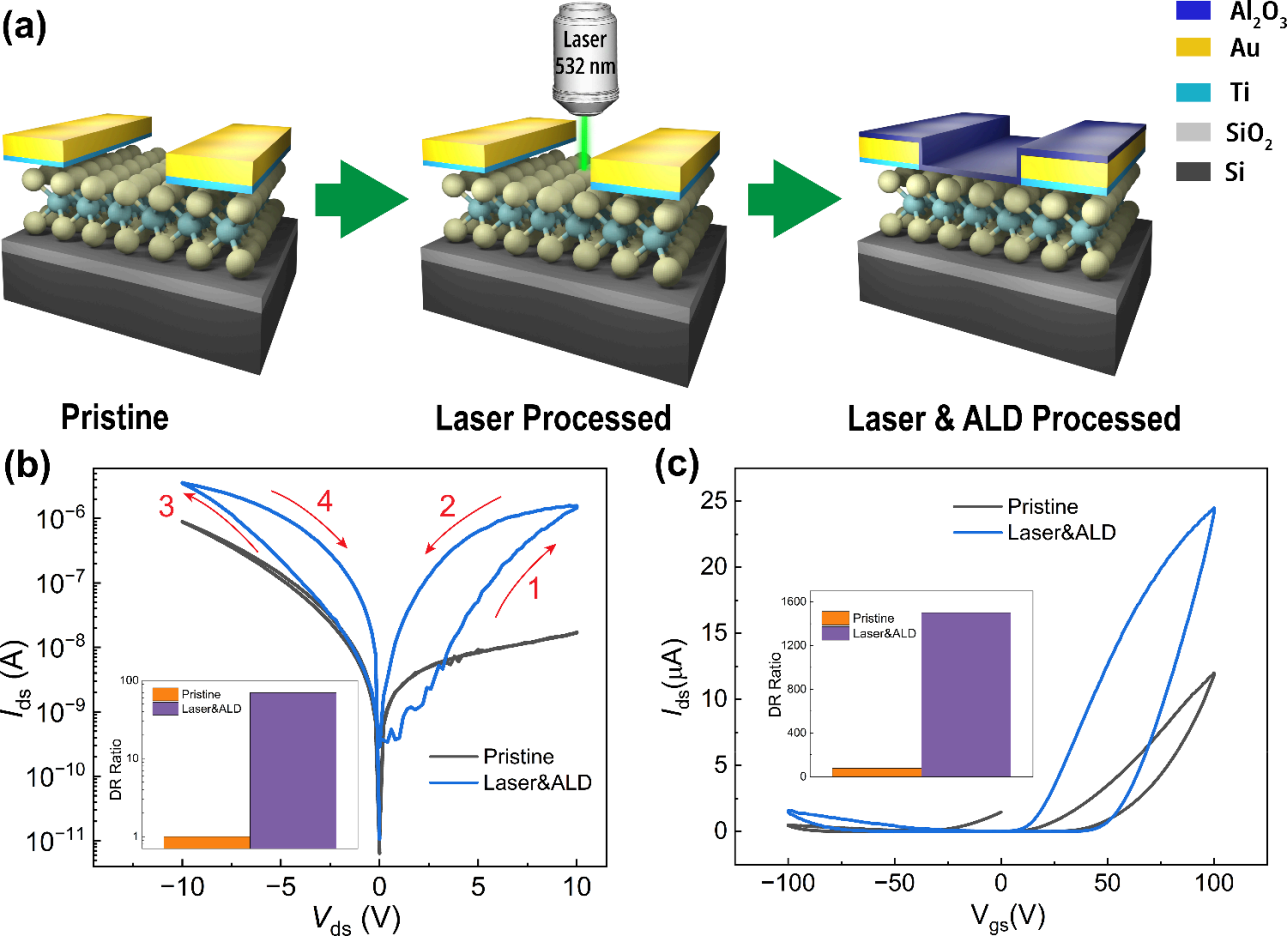
a) Schematic of pristine, laser processed, and laser and ALD processed MoTe_2_ devices. b) *I*_*ds*_ *–V*_*ds*_ characteristics of the pristine and processed (laser and ALD) MoTe_2_ memristors. The inset shows the dynamic range (DR) ratio for both memristors (DR calculated at *V*_*ds*_ = 2 V). c) *I*_*ds*_ *–V*_*gs*_ characteristics of the pristine and processed (laser and ALD) MoTe_2_ memtransistors at *V*_*ds*_ = 1 V. The inset shows the DR ratio for both memtransistors (DR calculated at *V*_*gs*_ = 25 V). The laser and ALD processed device was optically treated for 10 min with a 532 nm laser beam, followed by the ALD deposition of 20 nm of Al_2_O_3_ at 130 °C.

MoTe_2_ is reported as a 2D semiconductor with a bandgap of 1.1 eV.^23^ This relatively narrow bandgap makes it more sensitive to doping introduced by oxidation and gas absorption.^18,26^ As depicted in Figure 1b, the pristine device (15 nm thick) functioning as a memristor exhibits negligible hysteresis. In contrast, the same thickness device processed using a 532 nm CW laser for 10 min followed by ALD capping of a 20 nm Al_2_O_3_ layer deposited at 130 °C displays a remarkable hysteresis. The DR—defined as the resistance ratio between the high-resistance state (HRS) and the low resistance state (LRS)—increases by approximately 70-fold (calculated at *V*_ds_ = 2 V; see Figure 1b inset), and the maximum current improves by 4-fold. The same device can operate as a memtransistor by applying the gate voltage to the p-doped silicon substrate with the silicon dioxide (SiO_2_) layer serving as the gate dielectric. As illustrated in Figure 1c, the processed MoTe_2_ device operating as a memtransistor demonstrates a 20 times higher DR (calculated at *V*_gs_ = 25 V; see Figure 1c inset) and a 2.5 times higher maximum current compared to the pristine device. These findings for the 15 nm thick device are part of a broader investigation into the effects of the processing and material thickness. Three batches of samples with MoTe_2_ thicknesses ranging from 8 to 30 nm were prepared. The first batch was treated with laser irradiation (laser processed), the second batch was capped with 20 nm Al_2_O_3_ deposited by ALD (ALD processed), and the third batch was laser-treated before being covered with 20 nm ALD deposited Al_2_O_3_ (laser and ALD processed). See Supporting Information Section 2 for detailed electrical characterization of each process step at various material thicknesses.

It has been reported that optical treatment with 532 nm laser power of 9 mW or higher can induce structural transformations to MoTe_2_.^27,28^ These structural changes have been attributed to two distinct causes: a phase change from the 2H phase into the 1T′ phase^29,30^ or clustering of the MoTe_2_ tellurium atoms.^31,32^ In our study, we used a lower laser power of 5 mW to avoid inducing such a phase change or tellurium clustering during the optical treatment, which might compromise device performance. The Raman spectra of the pristine, laser, and laser and ALD processed MoTe_2_ flake are shown in Figure 2a. Pristine MoTe_2_ exhibits three prominent Raman modes at 170, 235, and 289 cm^–1^, identified as the *A*_*g*_^1^, *E*_2*g*_^1^, and *B*_2*g*_ modes, respectively. After 10 min of optical treatment, all Raman modes remain unchanged, indicating that MoTe_2_ retains its 2H crystalline phase with no noticeable evidence of the 1T′ phase (modes at 159 cm^–1^ ^31^) or tellurium clustering (modes at 120 cm^–1^ ^28^).

**Figure 2:**
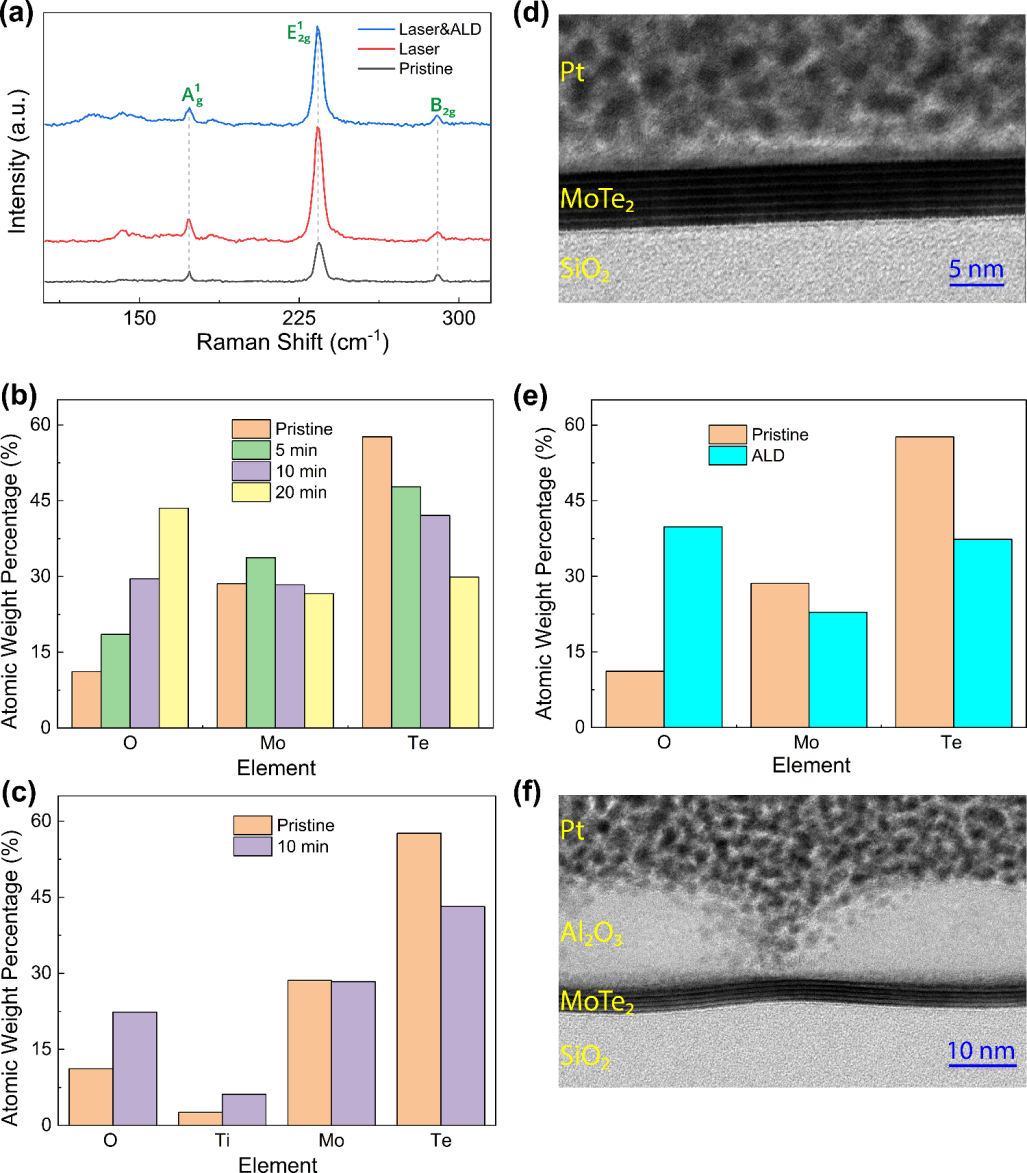
a) Raman spectra of pristine, laser processed, and laser and ALD processed MoTe_2_. b) Energy dispersive X-ray (EDX) spectroscopy results of laser processed MoTe_2_ under different irradiation times. c) EDX spectroscopy results of MoTe_2_ under the edge of electrodes in pristine and 10 min laser processed devices. d) Transmission electron microscopy (TEM) image of laser processed MoTe_2_. e) EDX spectroscopy results of ALD processed MoTe_2_. f) TEM image of ALD processed MoTe_2_.

Laser irradiation in an ambient environment facilitates oxidation of crystalline MoTe_2_ to amorphous MoO_*x*_ (*x* < 3). The increment in surface roughness of the flake measured by AFM suggests the top layer oxidization into MoO_*x*_ ^33^ (see Figure S12a). The optical image of the treated flake appears darker as the increased roughness leads to scattering of incident light in arbitrary directions^27^ (see Figure S12b). To confirm the formation of MoO_*x*_, cross-sectional transmission electron microscopy (TEM) and energy-dispersive X-ray spectroscopy (EDX) are employed. The EDX results, presented in Figure 2b for pristine and laser-treated MoTe_2_ (5, 10, and 20 min), show a decrease in the atomic concentration of tellurium from approximately 60% to 30%. This decrease is accompanied by a corresponding increase in oxygen concentration, indicating the replacement of tellurium by oxygen and, thus, the formation of molybdenum oxide. This oxidation-induced structure change has been previously observed in TMDs.^12,25^

Electrical measurements of all devices functioning as memristors reveal a significant increase in current with laser irradiation before reaching saturation (see Supporting Information Section 2 for more details). This current increment can be driven by two key mechanisms: p-type doping of the MoTe_2_ channel and improved metal–MoTe_2_ contacts. Oxidized MoTe_2_ acts as acceptors to introduce p-type doping, as evidenced by the memtransistor’s transfer characteristics (see Figure S6). Simultaneously, laser-induced annealing and oxide growth beneath the edge of the metal contact suppress the Schottky barrier, enhancing hole tunneling into the channel.^33^ See Supporting Information Section 4 for transfer length method (TLM) analysis showing suppression of the contact barrier after processing. Presented in Figure 2c, the EDX results for MoTe_2_ under the edge of the metal contact in pristine and 10 min laser-treated devices show an increase in oxygen concentration, which indicates oxide growth beneath the metal contact. Notably, the observed current saturation after a certain irradiation time is likely due to a self-limiting oxidation process that oxidizes only the top few layers of MoTe_2_, while preserving the 2H phase of the underlying layers. This structural stability is supported by Raman spectroscopy (see Figure 2a), which displays 2H MoTe_2_ peaks, and TEM imaging, which confirms uniform MoTe_2_ layers after treatment (see Figure 2d).

The transfer characteristics of all ALD processed devices functioning as memtransistors showed a transition toward n-type behavior (see Supporting Information Section 2 for more details). In our study, the ALD process uses water and trimethylaluminum (TMA) as precursors to deposit 20 nm of Al_2_O_3_ on top of MoTe_2_ at a temperature of 130 °C. During this process, several factors can contribute to the n-doping of the MoTe_2_ lattice. For example, the diffusion of hydrogen atoms can act as donors, or methyl groups dissociated from the TMA precursor can also transfer electrons.^34,35^ Consequently, the Fermi level shifts near the conduction band minimum, enhancing n-type conductivity. Simultaneously, the ALD process appears to introduce interface traps between the Al_2_O_3_ and MoTe_2_, likely due to residual oxygen and water molecules.^36–39^ See Supporting Information Section 5 for trap density calculation for pristine and processed (laser and ALD) devices. These traps contribute to a reduction in maximum current and an increase in hysteresis observed in the characteristics of memristors. EDX results of the ALD-processed MoTe_2_ confirm a significant increase in oxygen atomic concentration compared to the pristine material as shown in Figure 2e. TEM imaging reveals uniform MoTe_2_ layers beneath the deposited film and shows the formation of Al_2_O_3_ islands on top of MoTe_2_ (see Figure 2f). This island formation is consistent with previous reports.^40–42^

In the two-step process involving laser treatment followed by ALD, p- and n-type doping explain the changes in maximum current and majority charge carrier polarity but do not fully account for the observed DR enhancement. Notably, laser-treated devices exhibit excess surface oxygen, which increases the density of acceptor-like trap sites at the Al_2_O_3_/MoTe_2_ interface during the ALD process.^43^ The presence of these trap sites contributes to the enhanced hysteresis observed in the electrical characteristics of the memristor devices. Furthermore, to investigate the conduction mechanism in the processed (laser and ALD) memristor, double-logarithmic *I*–*V* curves are plotted for forward and reverse bias regions (see Supporting Information Section 6).

The controllable analogue switching behaviors of the processed memristor and memtransistor over device-to-device and cycle-to-cycle ensure the practicality of the proposed approach for high-performance neuromorphic applications (Figures S13 to S17). Operating as a memristor and a memtransistor, the processed device is capable of emulating homosynaptic and heterosynaptic plasticity. Figure 3a illustrates the basic structure of a biological synapse featuring homosynaptic plasticity, comprising the presynaptic membrane, neurotransmitters, and postsynaptic receptors.^44^ When the cell is stimulated, vesicles drift to the presynaptic membrane, where neurotransmitters are released into the synaptic cleft. These neurotransmitters are then received by postsynaptic receptors. The efficiency of this transfer process is expressed as the synaptic weight.^45^ Over time, the synaptic weight between two neurons changes based on synaptic activity: more active synapses strengthen through potentiation, whereas less active ones weaken through depression. By analogy, in a memristor-based artificial synapse, the left and right electrodes act as the pre- and postsynaptic neurons, respectively, and the memristor’s conductance represents the synaptic weight. This artificial weight can be tuned via voltage pulses, mimicking plasticity in a biological synapse.

**Figure 3:**
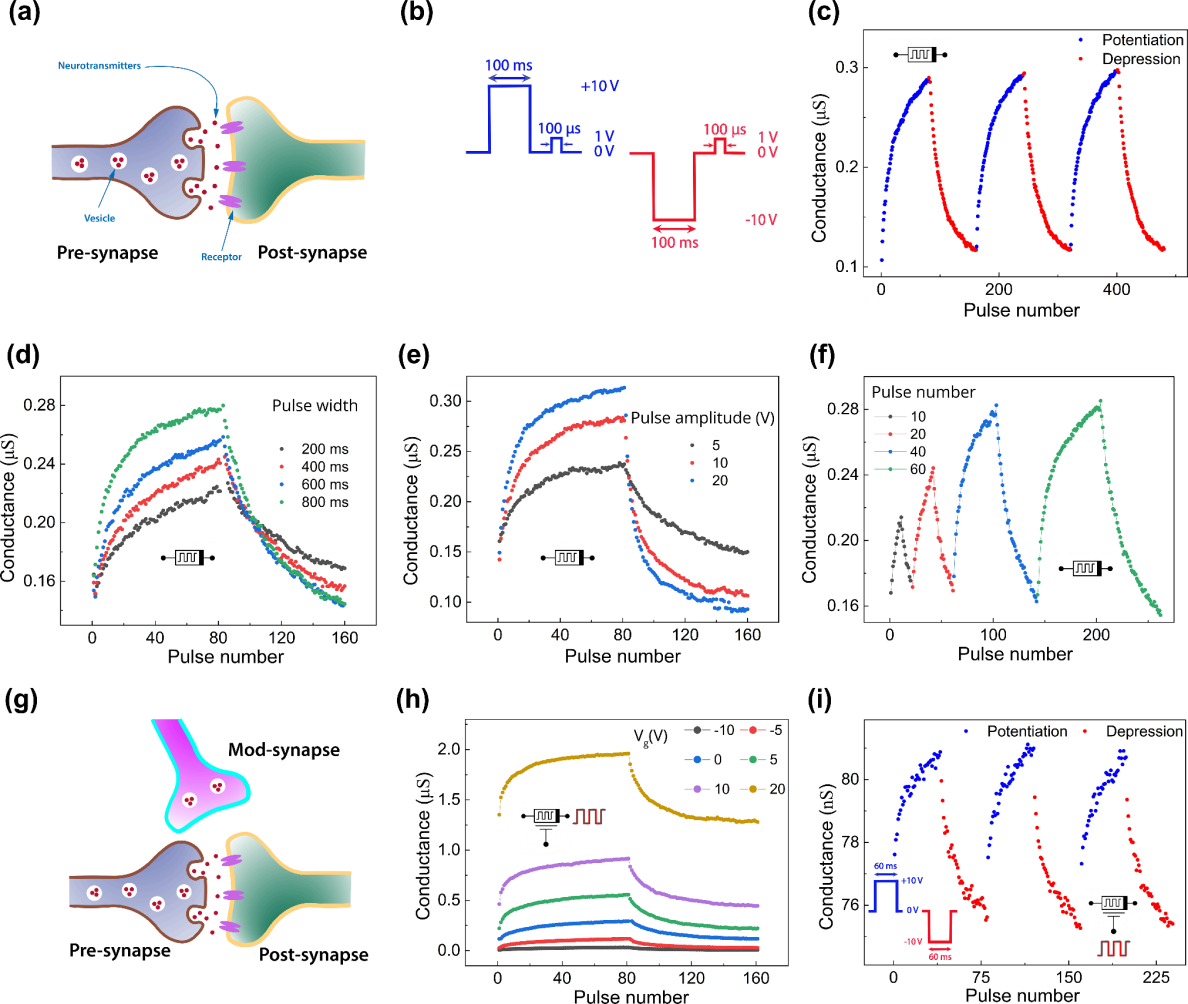
a) Schematic of a biological synapse with homosynaptic plasticity. b) Signal profile of the memristor’s applied voltage pulses. c) Manipulation of the memristor conductance by applying pulses shown in (b). Analogue switching behavior at different d) pulse width, e) pulse amplitude, and f) pulse number. g) Schematic illustration of heterosynaptic plasticity. h) Manipulation of the memtransistor conductance using the drain terminal as the presynaptic input by applying pulses shown in (b) under different gate voltages. (i) Analogue switching behavior of the memtransistor using the gate terminal as the presynaptic input at *V*_d_ = 1 V. The inset shows the applied signal profile.

Figure 3b displays two separate learning protocols on the left and right sides. Repeating the left protocol 80 times leads to an increase in the conductance of the memristor—representing synaptic strengthening—shown by the blue curves in Figure 3c. This corresponds to potentiation in a biological synapse. On the contrary, when the learning protocol on the right side of Figure 3b is repeated 80 times, the resulting conductance decrement is presented by the red curves in Figure 3c. This decrease in conductance corresponds to depression in the biological synapse, indicating a weakening of the synapse. These potentiation and depression behaviors have been repeated over 3 cycles (see Figure S18a for 9 cycles of potentiation and depression and Figure S19a for endurance).

The conductance of electronic synapses is significantly influenced by the characteristics of the input pulses. We investigated the impact of pulse parameters such as pulse width (200–800 ms, shown in Figure 3d), amplitude (5, 10, and 20 V, shown in Figure 3e), and number (10–60 pulses, shown in Figure 3f). These results clearly indicate that increasing the pulse width or amplitude leads to a faster rate of potentiation/depression and a larger DR. Additionally, increasing the number of pulses enhances the DR. Beyond potentiation and depression, we further studied the synaptic plasticity in our memristor by demonstrating the spike-timing-dependent plasticity (STDP) (Figure S20). Furthermore, to estimate the lowest energy consumption, pulses with an amplitude of ± 3V and a width of 100 μs were applied, resulting in an energy consumption of 12 pJ (Figure S21).

While homosynaptic plasticity considers only the changes at directly stimulated synapses, heterosynaptic plasticity involves modifications at nearby synapses, which are not directly involved in the initial activity (illustrated biologically in Figure 3g). To emulate heterosynaptic plasticity, either the drain or gate electrode can serve as the presynaptic membrane (input terminal for pulses), while the source electrode acts as the postsynaptic membrane. The device’s overall conductance can be altered by applying either positive or negative pulses.

When using the drain as the presynaptic membrane, applying the learning protocol shown in Figure 3b to the drain, while the gate electrode is modulated by a DC voltage between −10 and 20 V, leads to analogue switching depicted in Figure 3h. In addition to exhibiting potentiation and depression under different gate voltages, the device demonstrates dynamic modulation of its synaptic behavior, where negative gate voltages reduce its DR and positive gate voltages enlarge it.

The memtransistor’s conductance is also adjustable when the gate terminal acts as the presynaptic membrane. A train of 40 consecutive positive pulses (+10 V amplitude, 60 ms width) yields the blue curves shown in Figure 3i, whereas 40 negative pulses (−10 V amplitude, 60 ms width) result in the red curves. Applying positive pulses gradually increases the channel conductance, analogous to synaptic potentiation, while applying negative pulses gradually decreases the conductance, mirroring synaptic depression. These potentiation and depression behaviors have been repeated over 3 cycles (see Figure S18b for 9 cycles of potentiation and depression and Figure S19b for endurance).

Finally, to evaluate the practical learning capabilities of both processed and pristine devices, we conducted an artificial neural network (ANN) simulation using the CrossSim simulator. The learning performance was assessed through the recognition rate of a binarized handwritten digit data set from the Modified National Institute of Standards and Technology (MNIST).^46^

As shown in Figure 4a, the three-layer ANN incorporates 784 neurons in its input layer, configured to accept 28 × 28 pixel MNIST images. This data then pass to a hidden layer containing 300 neurons. For the final digit classification, the output layer utilizes 10 neurons with each representing a distinct digit class (0–9). The simulation was conducted based on experimentally measured data in Figure 4b, and the recognition rate was evaluated using a data set of 60,000 training images and 10,000 test images.

**Figure 4:**
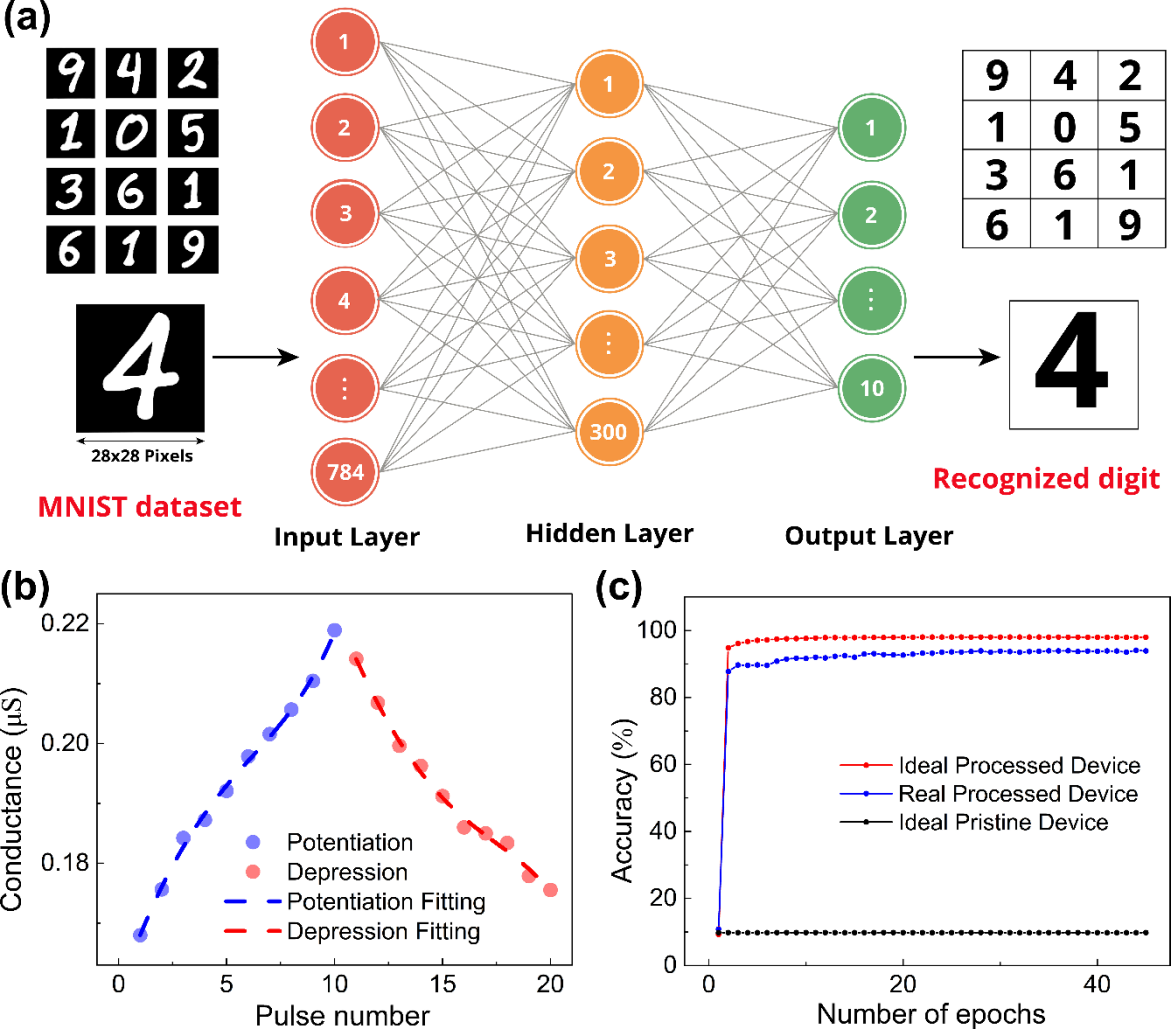
a) Schematic of a three-layer ANN with 784 input neurons, 300 hidden neurons, and 10 output neurons. b) Potentiation and depression characteristics of the processed device with fitting. c) Recognition rates of ideal pristine, ideal processed, and real processed devices.

Figure 4c illustrates the recognition rate variation for the MNIST data set over 45 training epochs. For ideal devices exhibiting perfect linearity and symmetric potentiation and depression characteristics, the recognition rate for the processed device reaches 98%. By applying the experimentally derived nonlinearity (NL) values for the processed device (NLP = 1.13 for potentiation and NLD = −1.6 for depression), the recognition rate reaches about 94%, whereas the pristine device remains at approximately 9.8% (see Table S1 for an overview of recent advancements in processing techniques to enhance the performance of 2D-based memristors, comparing our work with others in terms of DR enhancement ratio, nonlinearity, energy, endurance, and ANN recognition percentage).

In conclusion, this study demonstrates an effective approach to expanding the memory window of 2D MoTe_2_, leveraging its full capacity for neuromorphic applications. This two-step approach involves an initial laser treatment, followed by an ALD deposition of Al_2_O_3_. The synergistic effects of both steps enhance the DR characteristics by introducing a high density of trap sites at the Al_2_O_3_/MoTe_2_ interface, resulting in significant performance improvements. When operating as a memristor, the processed device demonstrated a 70-fold improvement in DR ratio and a 4-fold increase in maximum current. As a memtransistor, it showed a 20-fold enhancement in DR ratio and a 2.5-fold improvement in maximum current. These improvements are crucial for the development of high-performance artificial synapses. Combining memristor and memtransistor functions in a single unit enables the processed device to emulate both homosynaptic and heterosynaptic plasticity, which are crucial for normal nervous system operation. Moreover, ANN simulations showed a 10-fold improvement in pattern recognition accuracy after 45 training epochs. This study provides a practical and impactful method for improving the performance of 2D neuromorphic devices.

## Supplementary Material



## Data Availability

Data will be made available on request.

## Notes

Experimental details, characterization methods, and figures presenting AFM and electrical measurement results (PDF)
