# Supplementary material for: Enhanced 2D MoTe2 Analogue Switching through Laser Processing and ALD-Passivation for Dual-Function Neuromorphic Devices
Source: Nano Lett. 2025 Dec 15;25(51):17970–7. doi: 10.1021/acs.nanolett.5c05915 (PMC12750991; doi:10.1021/acs.nanolett.5c05915)
Supplement: Supplementary file 1 [file nl5c05915_si_001.pdf]

Supporting Information

Enhanced 2D $MoTe_2$ Analogue Switching through Laser Processing and ALD-Passivation for Dual-Function Neuromorphic Devices

Mohamed Radwan[1,], Seyed Hossein Hosseini-Shokouh[1,*], Abde Mayeen Shafi[1], Catarina Dias[2], Fooqia Khalid[1],* Shreya Rajeevan[1], *Henri Hyttinen[1], Fida Ali[1], Faisal Ahmed[1], Zhipei Sun[1,3], and Harri Lipsanen[1,*]*

[1] Department of Electronics and Nanoengineering Aalto University, Tietotie 3 FI-02150, Finland.

[2] IFIMUP, Departamento de Física e Astronomia, Faculdade de Ciências, Universidade do Porto, Rua do Campo Alegre s/n, 4169-007 Porto, Portugal

[3] QTF Centre of Excellence, Department of Applied Physics Aalto University, Aalto FI-00076, Finland.

*Corresponding author: mohamed.radwan@aalto.fi, seyed.hosseinishokouh@aalto.fi, harri.lipsanen@aalto.fi

1. Experimental Details

Device fabrication

The fabrication process begins with the mechanical exfoliation of multilayer $MoTe_2$ flakes from bulk crystal (2D Semiconductors) using Scotch tape. These flakes are then transferred using the 2D transfer system (HQ Graphene) over a P-doped silicon substrate (0.001–0.005 Ω cm) covered with a 285 nm thick SiO_2 layer with pre-deposited gold markers. Next, electron beam lithography (EBL Vistec, EPBG 5000) defines the electrodes with a 5 µm channel width and a 3 µm channel length. The metal deposition takes place in an electron beam evaporator (MASA, IM-9912) under a chamber pressure of about 10^{-7} torr, where a 5 nm Ti adhesion layer is deposited followed by 50 nm of Au. Optical treatments are performed using a commercial WiTec Alpha300 RA+ Confocal Raman setup, equipped with a 532 nm CW laser operating at 5 mW and a ZEISS 100x (NA=0.9) objective. The treatments are done with the tool's image scan mode, which uses a raster pattern whose resolution is determined by lines per image and points per line. Around 20 nm of Al_2O_3 was grown on ALD tool (Beneq TFS-500) using TMA and water at 130 °C.

Characterization

The height profile of the $MoTe_2$ flake was measured with AFM (Bruker, Dimension Icon). The room-temperature Raman spectra of the exfoliated $MoTe_2$ were carried out using confocal micro-Raman (WITec Alpha300 RA+). A 20 nm platinum layer was deposited (Angstrom Engineering) to prepare the sample for lamella making (JEOL JIB-4700F). TEM and EDX are performed with JEOL JEM-2200 FS. All current-voltage measurements were carried out with a custom-built setup based on a Linkam LN600-P probe station using a source-measure unit (Keithley 2400). All pulse measurements were performed using Keysight B1500A Semiconductor Device Parameter Analyzer and tungsten microprobes.

2. Detailed Electrical Characterization for Each Step Process

Three batches of samples with $MoTe_2$ thicknesses ranging from 8 to 30 nm were prepared. The first batch was treated with laser irradiation (laser processed), the second batch was capped with 20 nm Al_2O_3 deposited by ALD (ALD processed), and the third batch was laser treated before being covered with 20 nm ALD deposited Al_2O_3 (laser and ALD processed). Electrical measurements indicated distinct behaviors based on thickness, leading to the categorization of samples into three groups: less than 10 nm, between 10, and 25 nm, and more than 25 nm.

We investigated the impact of laser illumination on the electrical characteristics of memristors and memtransistors across each thickness category. The key optical treatment parameters include laser power, irradiation time, scan area, separation between irradiation spots, and the number of spots. With the laser power, scan area, and separation between irradiation spots set at 5mW, 36 μm^2, and 0.01 µm, respectively, the total irradiation time is determined by the number of irradiation spots. The irradiation duration was set to 5, 10, 15, 20, 25, and 35 min.

For memristor operational mode, the IV characteristics of all laser-processed devices showed a maximum current increase after a certain period of laser irradiation, after which the current saturated. In devices with thicknesses around 8, 10, and 15 nm, the maximum current increased by nearly 2 orders of magnitude After 5, 10, and 20 min of laser irradiation, respectively, (see Figure S2 for 8 nm device, Figure S3 for 10 nm device, and Figure S4 for 15 nm device). In contrast, the device with thickness around 30 nm showed only 1 order of magnitude increase after 5 min; this thicker device appears to require more than 35 min to reach saturation (see Figure S5). However, none of the laser treated devices showed significant improvement in hysteresis.

For memtransistor mode, the transfer characteristics of all laser-processed devices showed a transition towards P-type behavior. The 8 and 15 nm thick $MoTe_2$ devices shifted from ambipolar and N-type behaviors, respectively, towards P-type behavior (see Figures S6a and S6b, respectively). For the 30 nm thick device, the pristine material showed N-type dominant behavior. Although laser irradiation induced a shift toward ambipolarity, 35 min of treatment was not sufficient for a complete transition to P-type (see Figure S6c).

Laser treatment was observed to enhance device current without improving hysteresis. Given that ALD can introduce trap sites at the $Al_2O_3/MoTe_2$ interface, we investigated capping of pristine devices with a 20 nm Al_2O_3 layer to address the limited DR enhancement. In all devices functioning as memristors, ALD capping resulted in a significant decrease in current and a notable enhancement in hysteresis (see Figure S7). In memtransistor mode, the transfer characteristics of all ALD processed devices showed a transition towards N-type behavior (see Figure S8).

Given that ALD treatment enhances hysteresis but reduces current, while laser processing improves current, a sequential approach was investigated to exploit their complementary

effects, aiming to synergistically enhance overall device performance. All devices were first treated with 10 min of laser processing, then capped with 20 nm Al_2O_3 deposited at 130 °C using ALD. In memristor operation, all devices exhibited an increase in current after laser processing. Subsequent ALD capping reduced the enhanced current while improving the hysteresis. It is worth mentioning that all laser and ALD processed devices showed higher current compared to their pristine state. While the 15 nm thick sample showed the most significant improvement in the DR ratio (see Figure S9a and S9b), the 8 and 30 nm thick devices displayed comparatively smaller hysteresis improvements (see Figures S10a and S11a, respectively). For memtransistor operation, Laser processing converted the 15 nm thick pristine N-type $MoTe_2$ device to P-type behavior. Subsequent ALD capping reverted the device back to N-type (see Figures S9c and S9d). The 8 nm thick memtransistor initially displayed ambipolar behavior, transitioning to P-type after light treatment, before reverting to ambipolar behavior following ALD capping (see Figure S10b). The 30 nm thick device began with N-type characteristics, shifted to ambipolar behavior, and then reverted to predominantly N-type behavior (see Figure S11b).

3. Supporting Figures

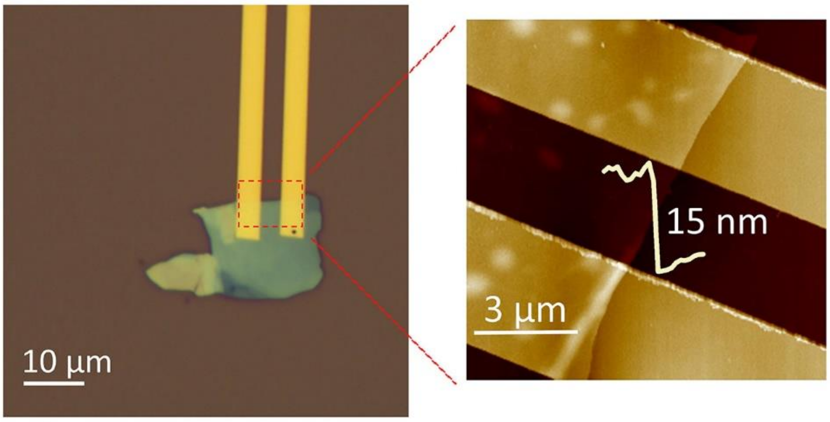

Figure S1. Optical microscopic image of the fabricated $MoTe_2$ device. The inset shows the AFM image with the height profile of the flake.

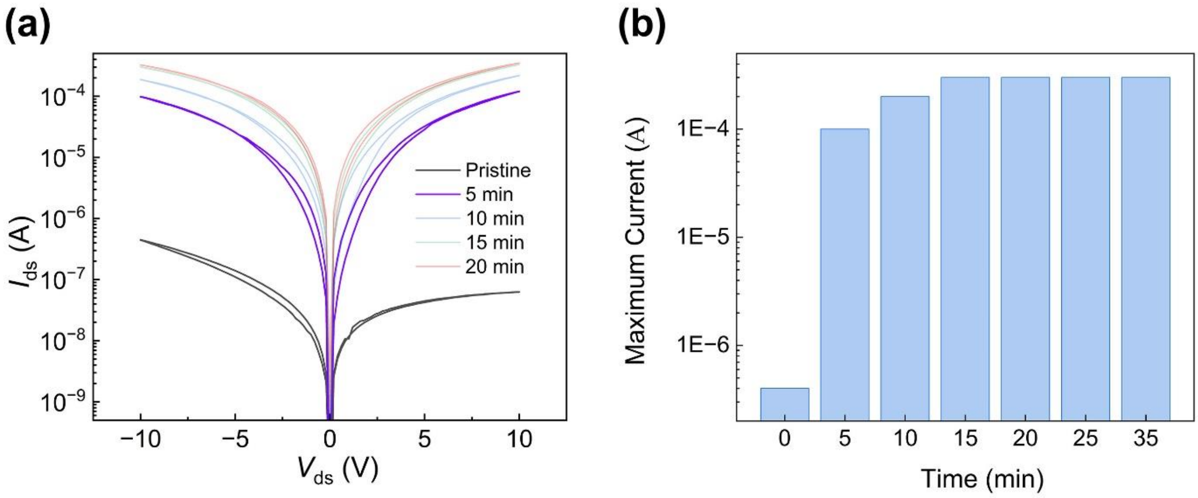

Figure S2. Characteristics of the pristine and laser processed $MoTe_2$ memristor with thickness around 8 nm. a) Electrical I_{ds}-V_{ds} characteristics. b) Variation in maximum current with irradiation time.

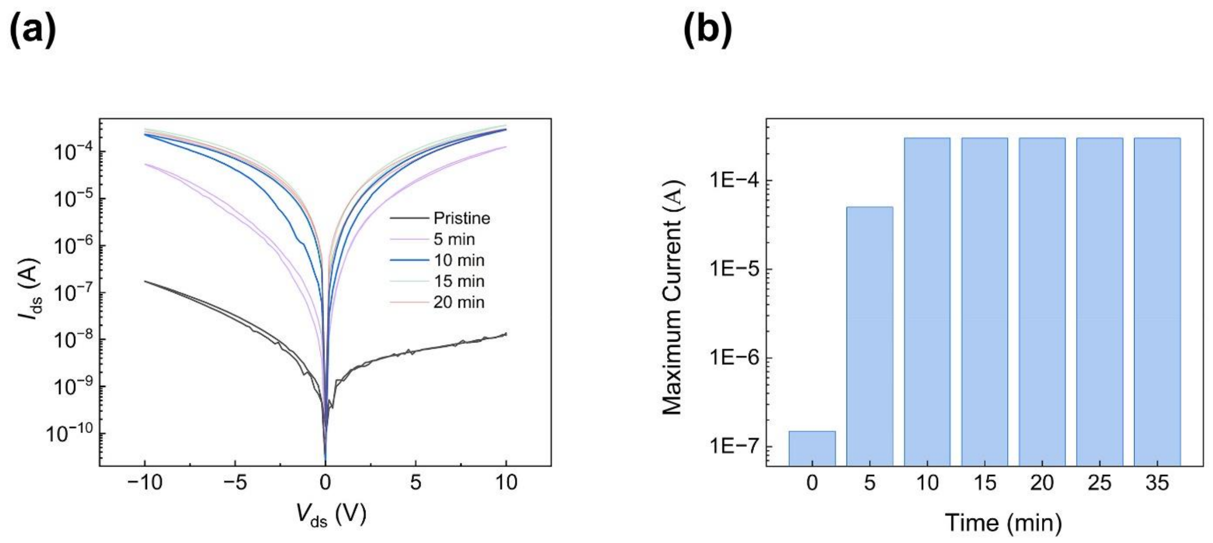

Figure S3. Characteristics of the pristine and laser processed $MoTe_2$ memristor with thickness around 10 nm. a) Electrical I_{ds}-V_{ds} characteristics. b) Variation in maximum current with irradiation time.

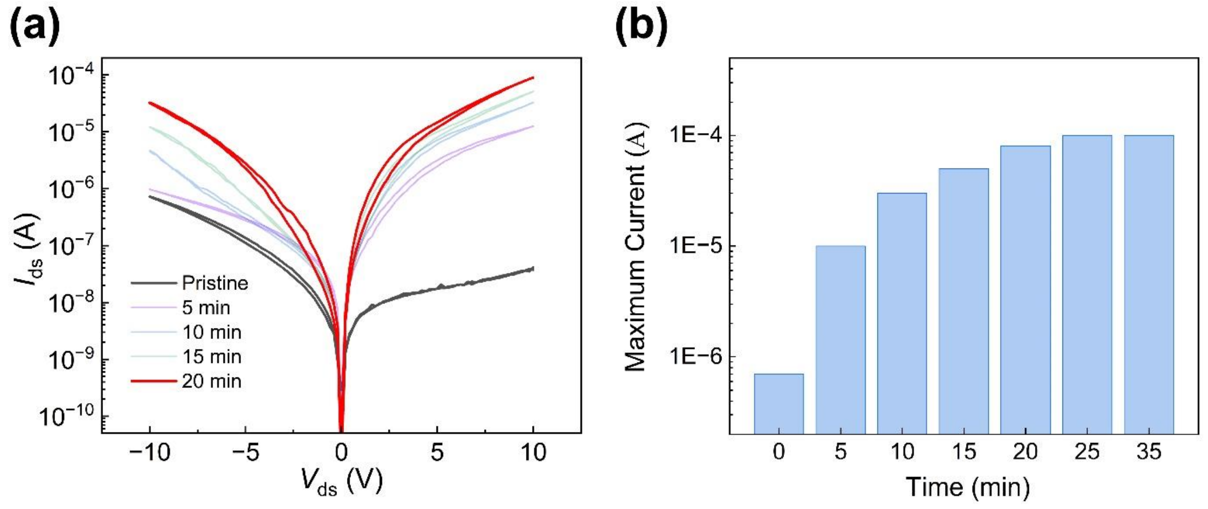

Figure S4. Characteristics of the pristine and laser processed $MoTe_2$ memristor with thickness around 15 nm. a) Electrical I_{ds}-V_{ds} characteristics. b) Variation in maximum current with irradiation time.

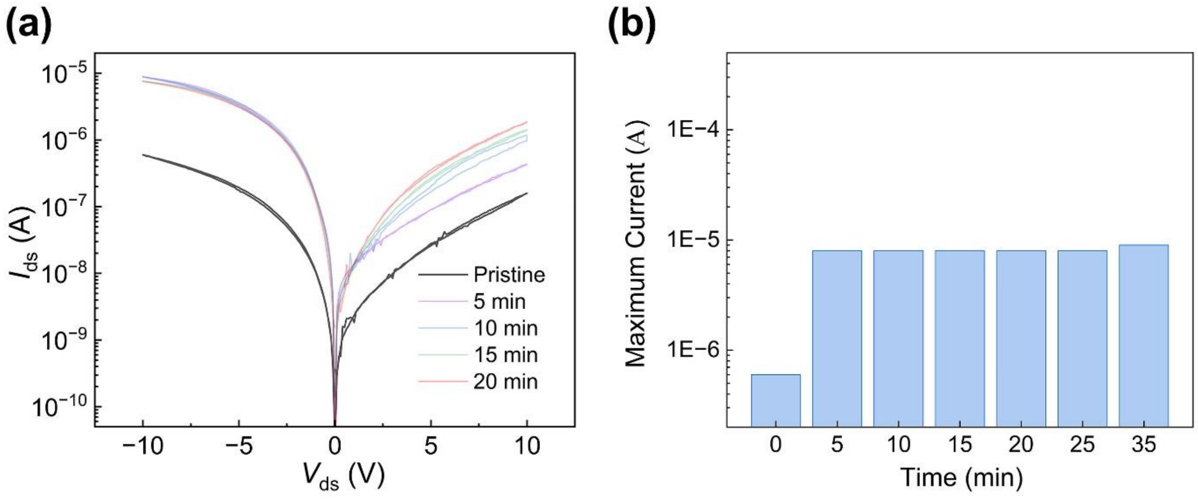

Figure S5. Characteristics of the pristine and laser processed $MoTe_2$ memristor with thickness around 30 nm. a) Electrical I_{ds}-V_{ds} characteristics. b) Variation in maximum current with irradiation time.

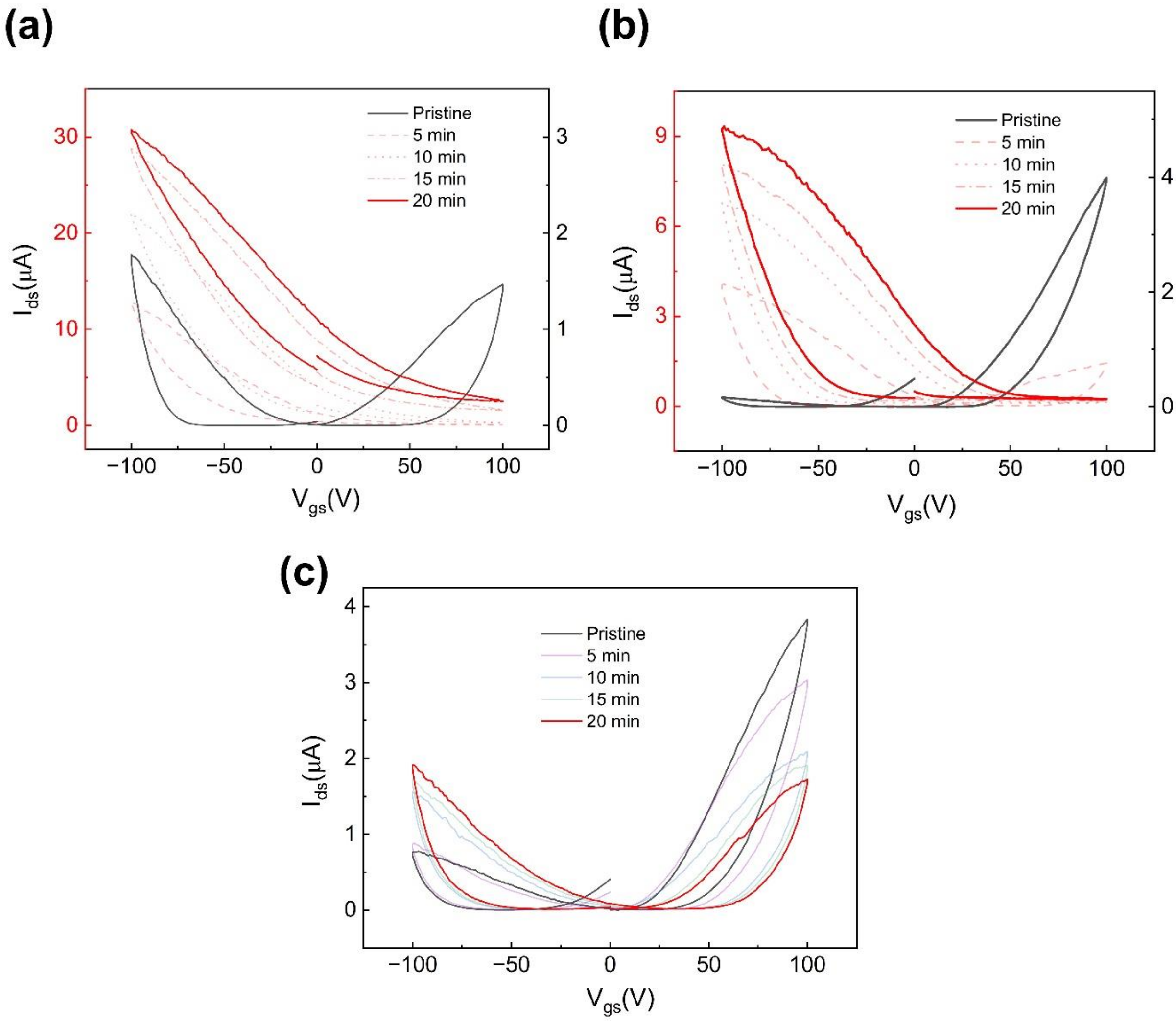

Figure S6. Electrical I_{ds}-V_{gs} characteristics of the pristine and laser processed $MoTe_2$ memtransistor at V_{ds}=|1| V with flake thickness around a) 8 nm, b) 15 nm, and c) 30 nm.

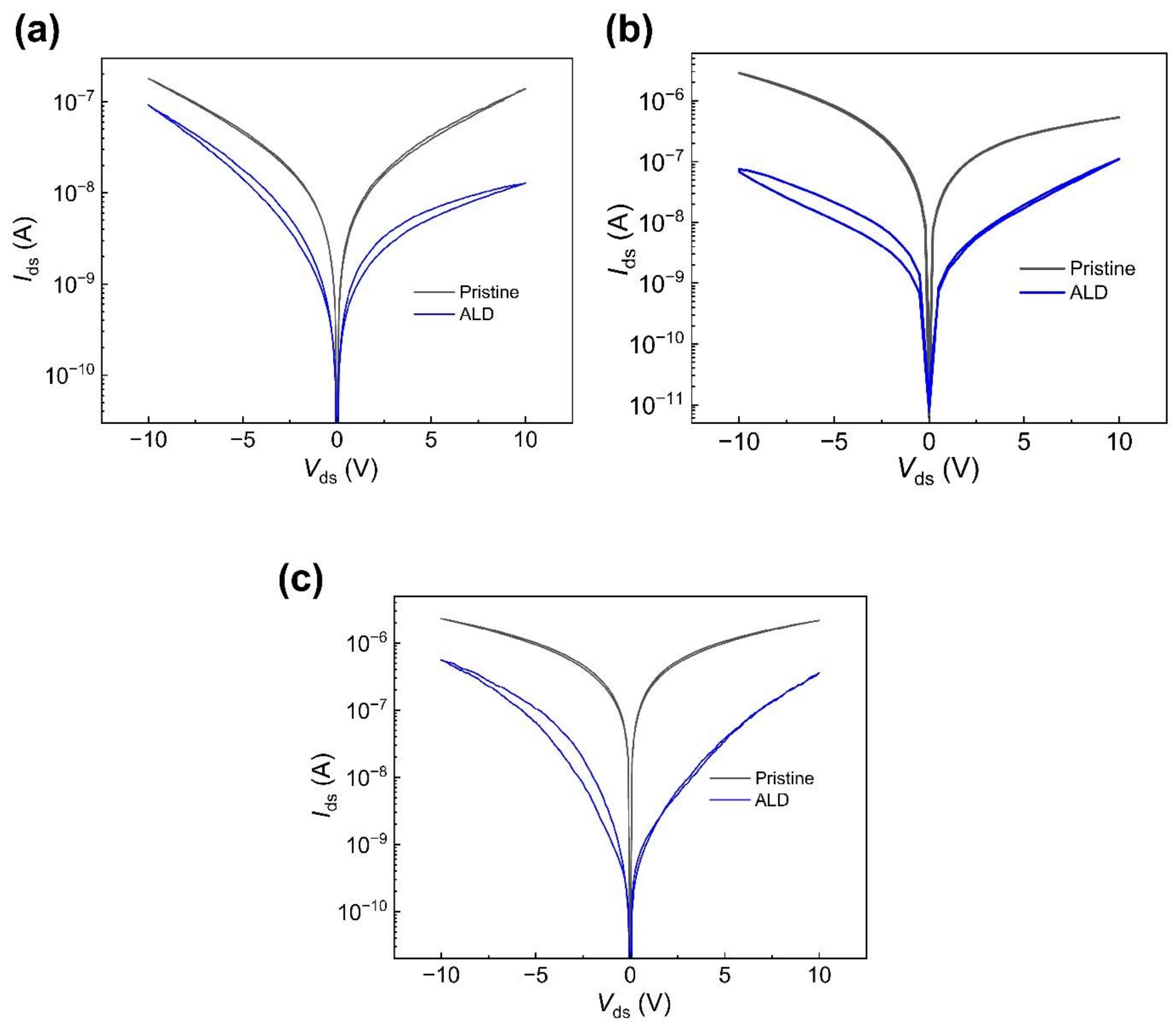

Figure S7. I_{ds}-V_{ds} characteristics of the pristine and Al_2O_3 capped $MoTe_2$ memristor with Flake thickness around a) 8 nm, b) 15 nm, and c) 30 nm.

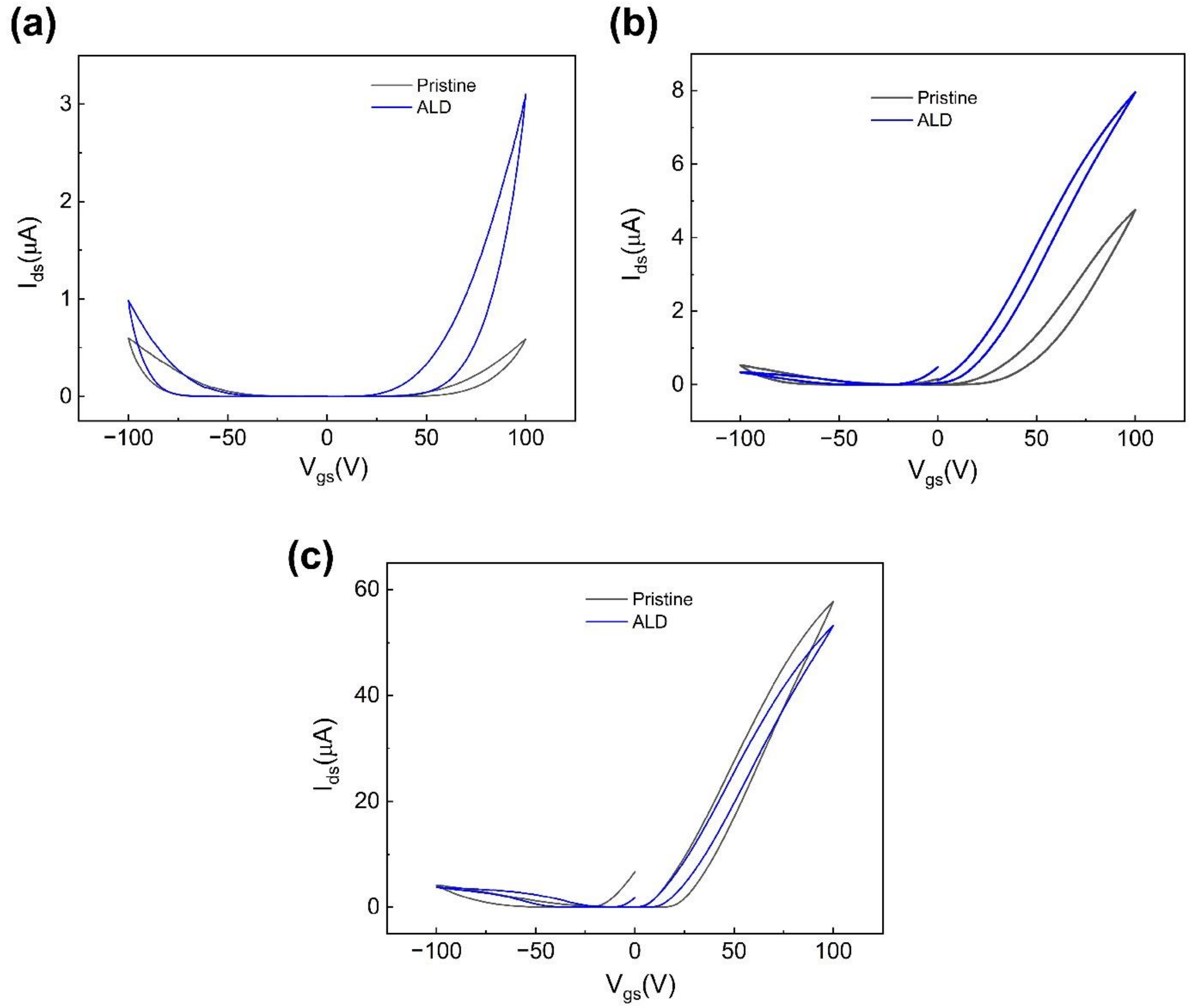

Figure S8. I_{ds}-V_{gs} characteristics of the pristine and Al_2O_3 capped $MoTe_2$ memtransistor with Flake thickness around a) 8 nm, b) 15 nm, and c) 30 nm.

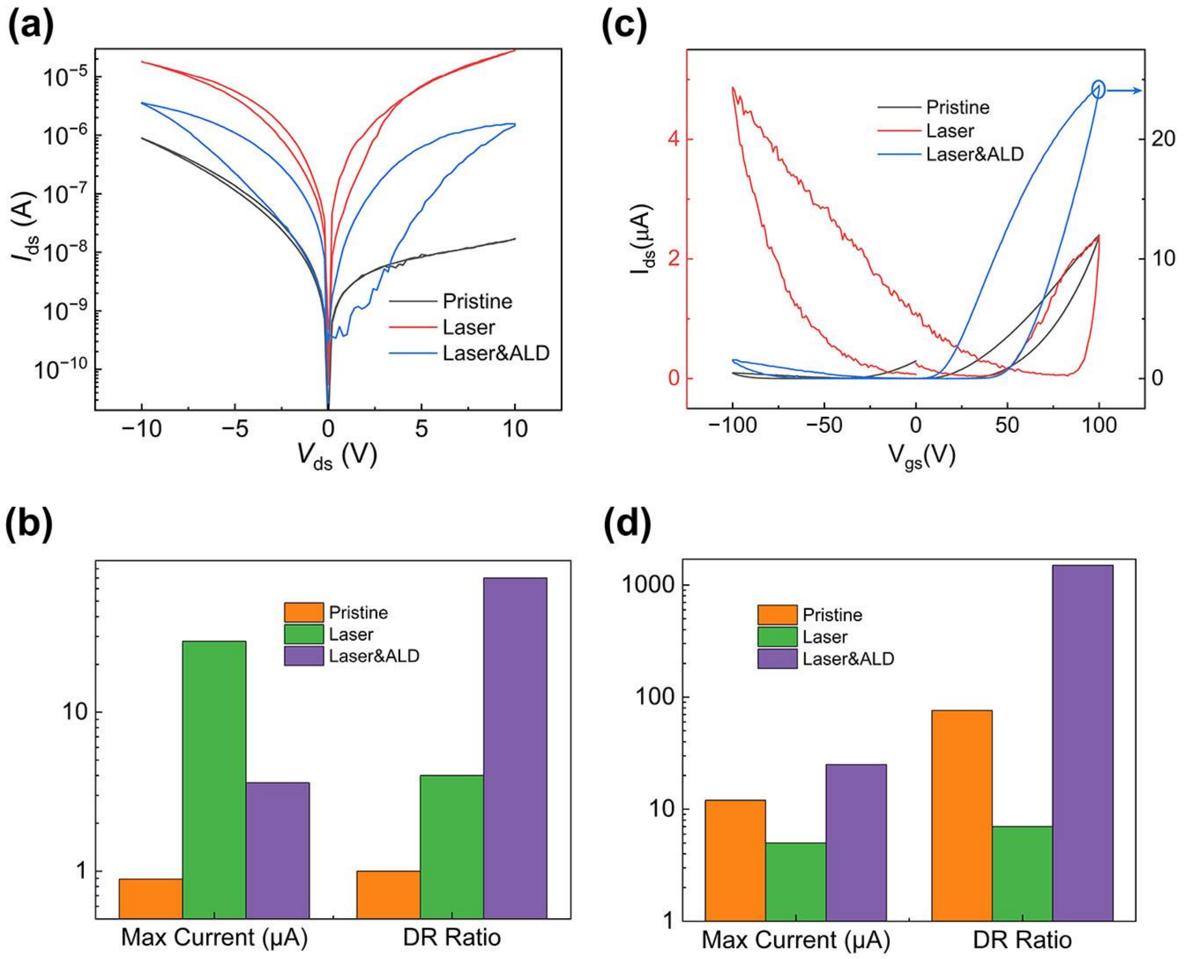

Figure S9. a) I_{ds}-V_{ds} characteristics of the pristine, laser treated, and laser and ALD processed memristor with thickness of 15 nm. b) Variation in maximum current and dynamic range (DR) ratio for pristine, laser treated, and laser and ALD processed $MoTe_2$ memristor (DR calculated at V_{ds} = 2 V). c) I_{ds}-V_{gs} characteristics of the pristine, laser treated, and laser and ALD processed memtransistor at V_{ds}=|1|V. d) Variation in maximum current and DR ratio for pristine, laser treated, and laser and ALD processed $MoTe_2$ memtransistor (DR calculated at V_{gs} = 25 V).

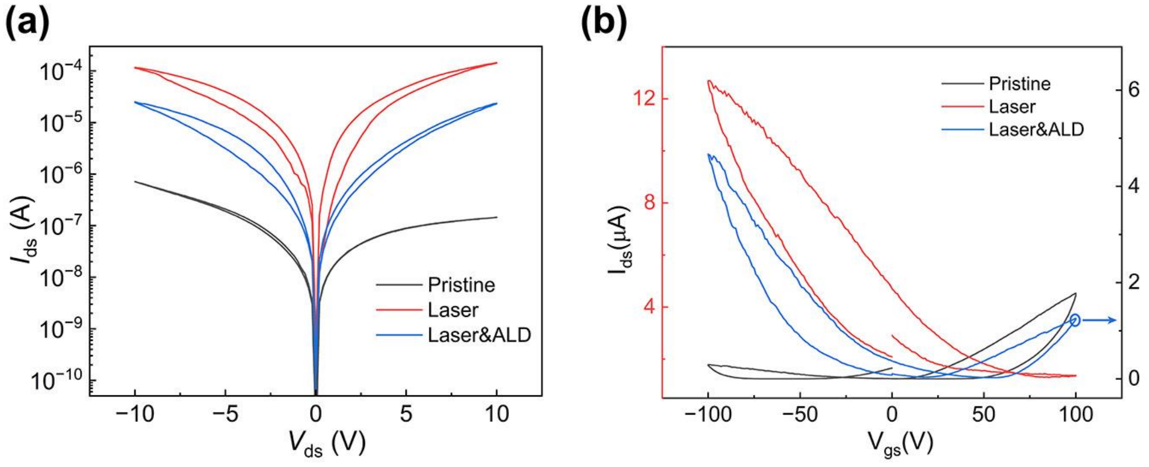

Figure S10. Electrical characteristics of the pristine, laser processed, and laser and ALD processed $MoTe_2$ device with thickness around 8 nm. a) I_{ds}-V_{ds} characteristics of memristor. b) I_{ds}-V_{gs} characteristics of the memtransistor at V_{ds}=|1|V.

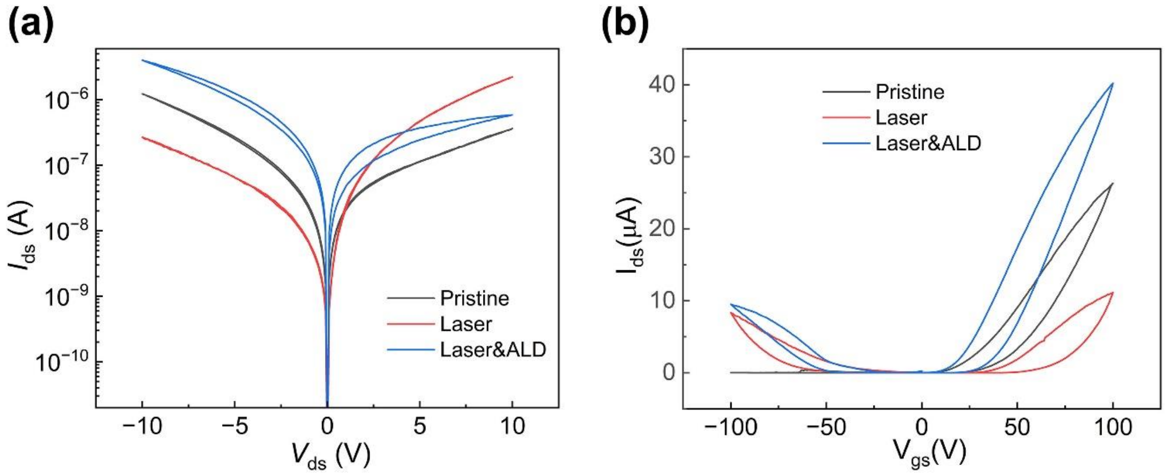

Figure S11. Electrical characteristics of the pristine, laser processed, and laser and ALD processed $MoTe_2$ device with thickness around 30 nm. a) I_{ds}-V_{ds} characteristics of memristor. b) I_{ds}-V_{gs} characteristics of the memtransistor at V_{ds}=|1|V.

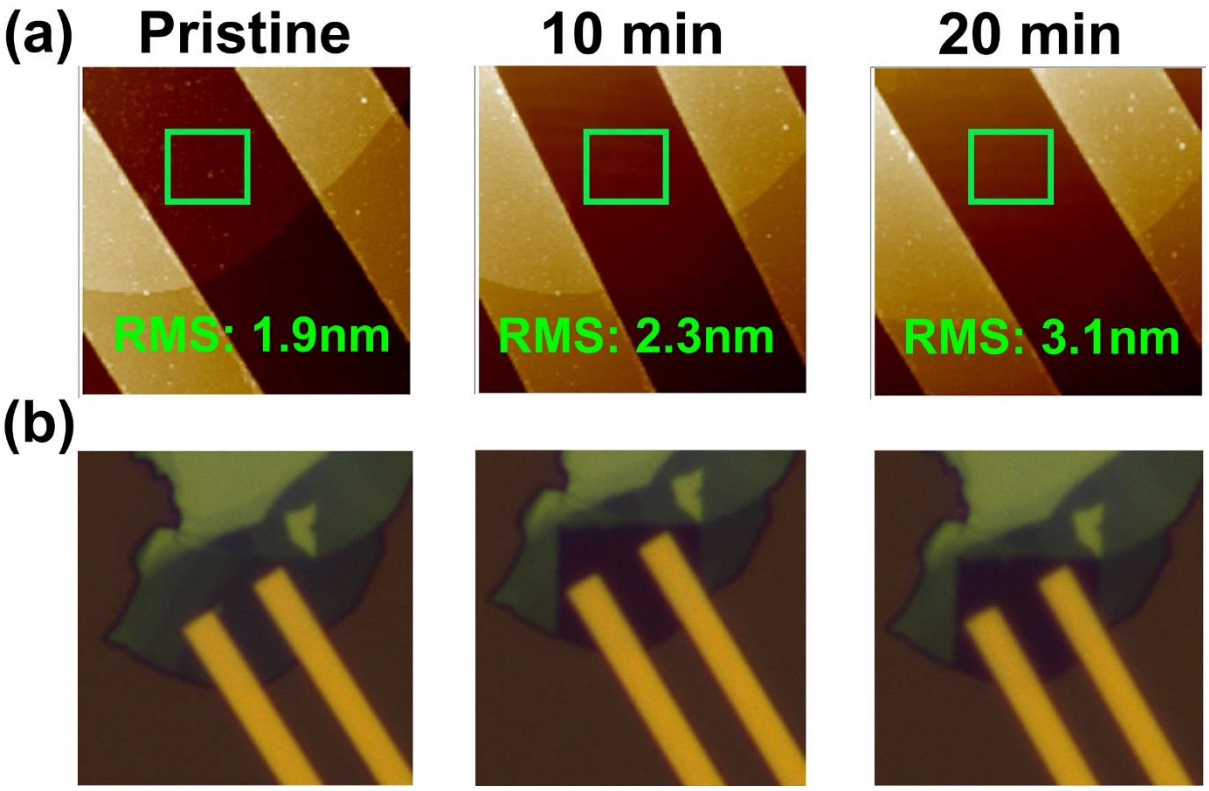

Figure S12. a) AFM images with corresponding RMS surface roughness for pristine and laser processed $MoTe_2$ flake. b) Corresponding optical microscopy images.

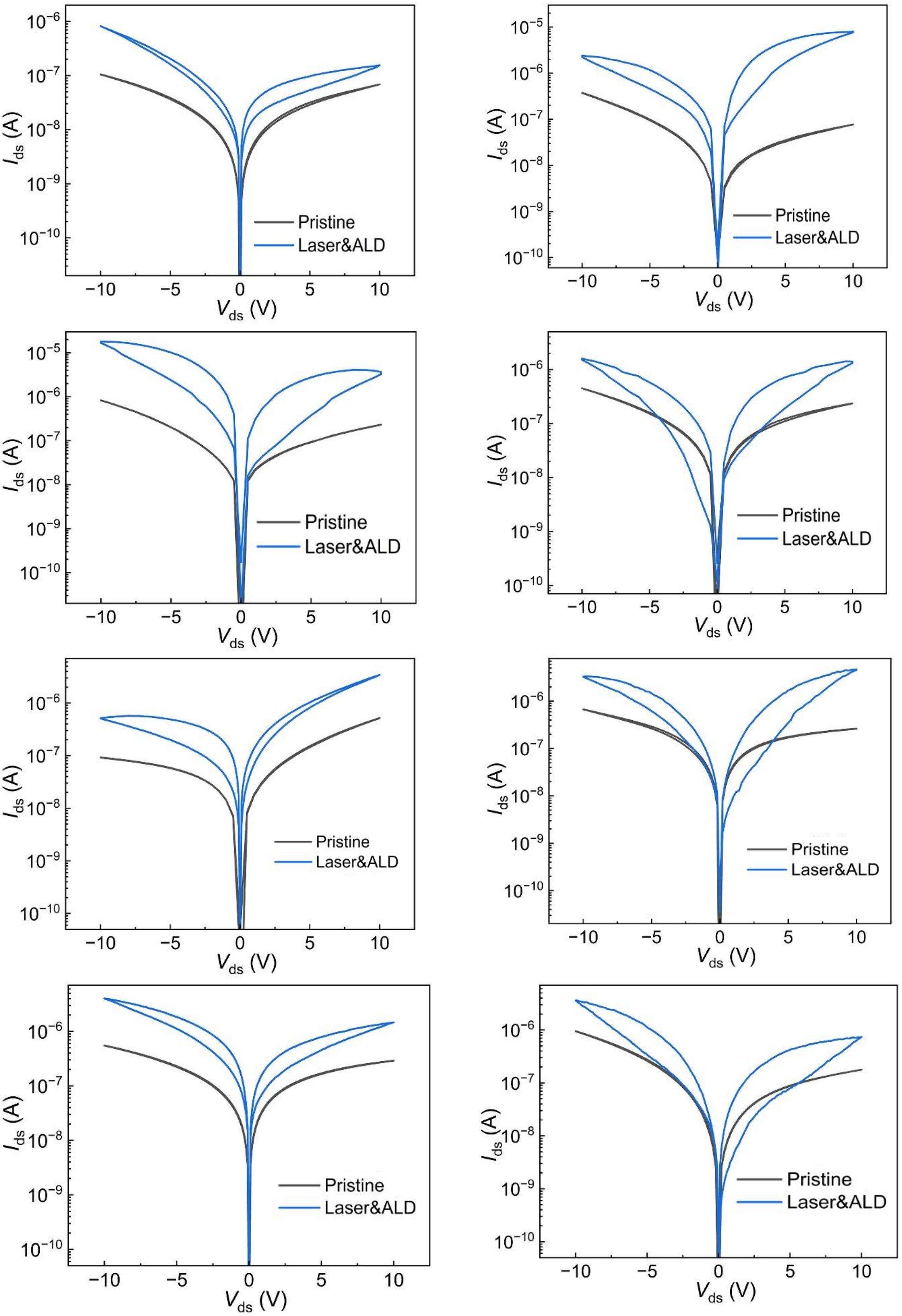

10^{-5}
10^{-6}
10^{-7}
10^{-8}
10^{-9}
10^{-10}
I_{ds} (A)
−10
−5
0
5
10
V_{ds} (V)
Pristine
Laser&ALD

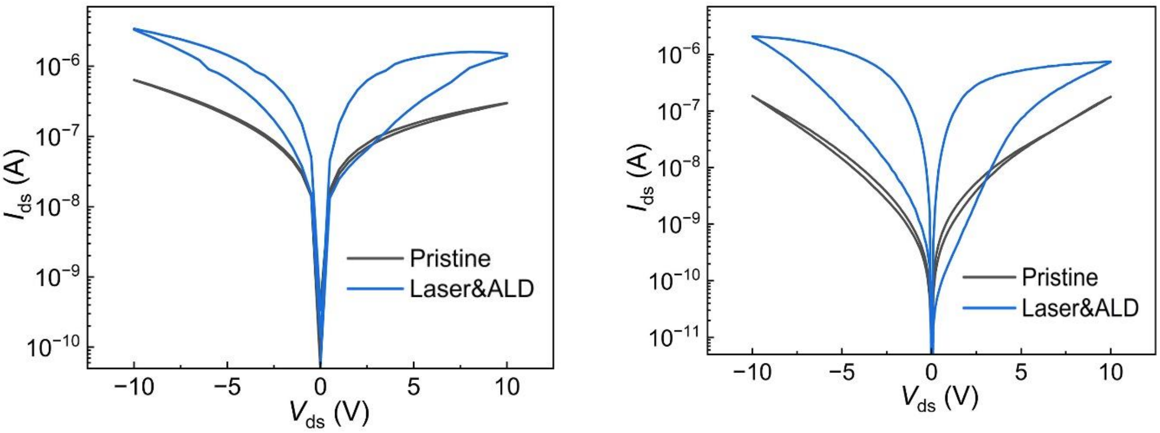

Figure S13. Electrical I_{ds}-V_{ds} characteristics of the pristine and processed (laser and ALD) $MoTe_2$ memristors for different devices.

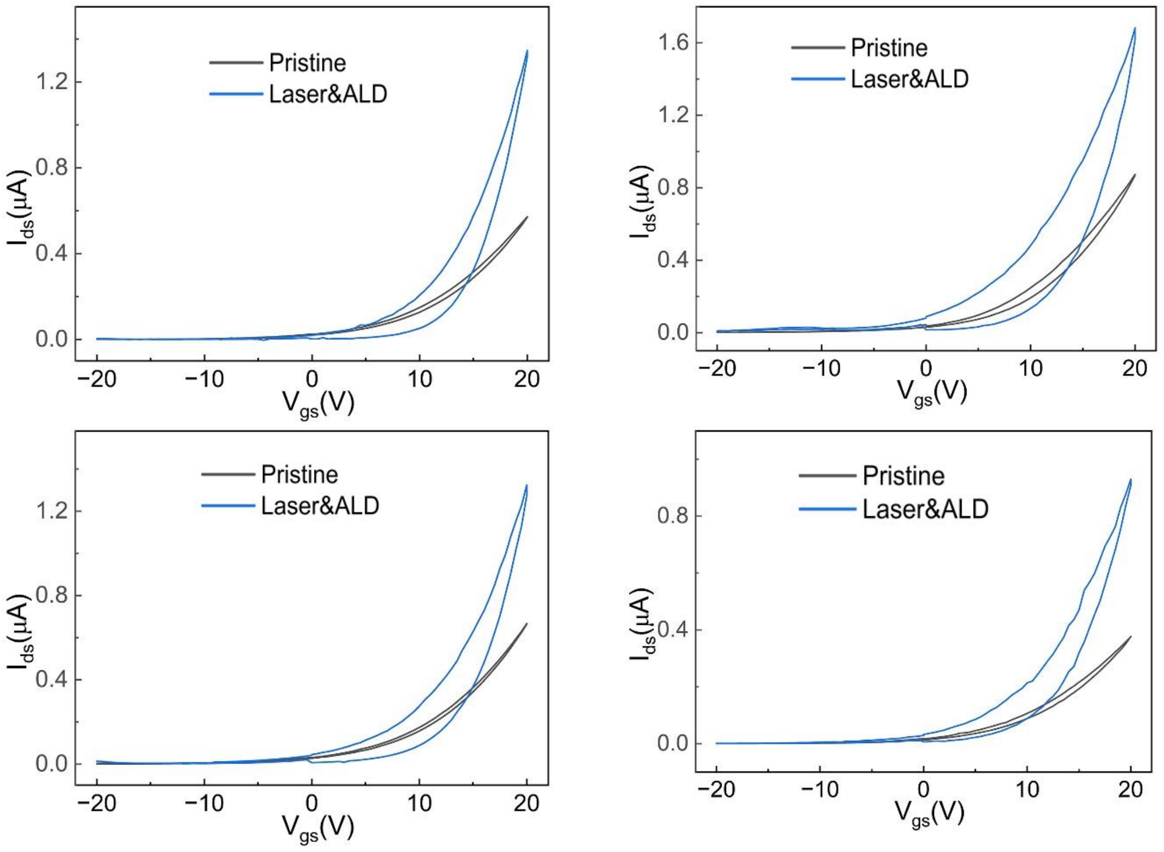

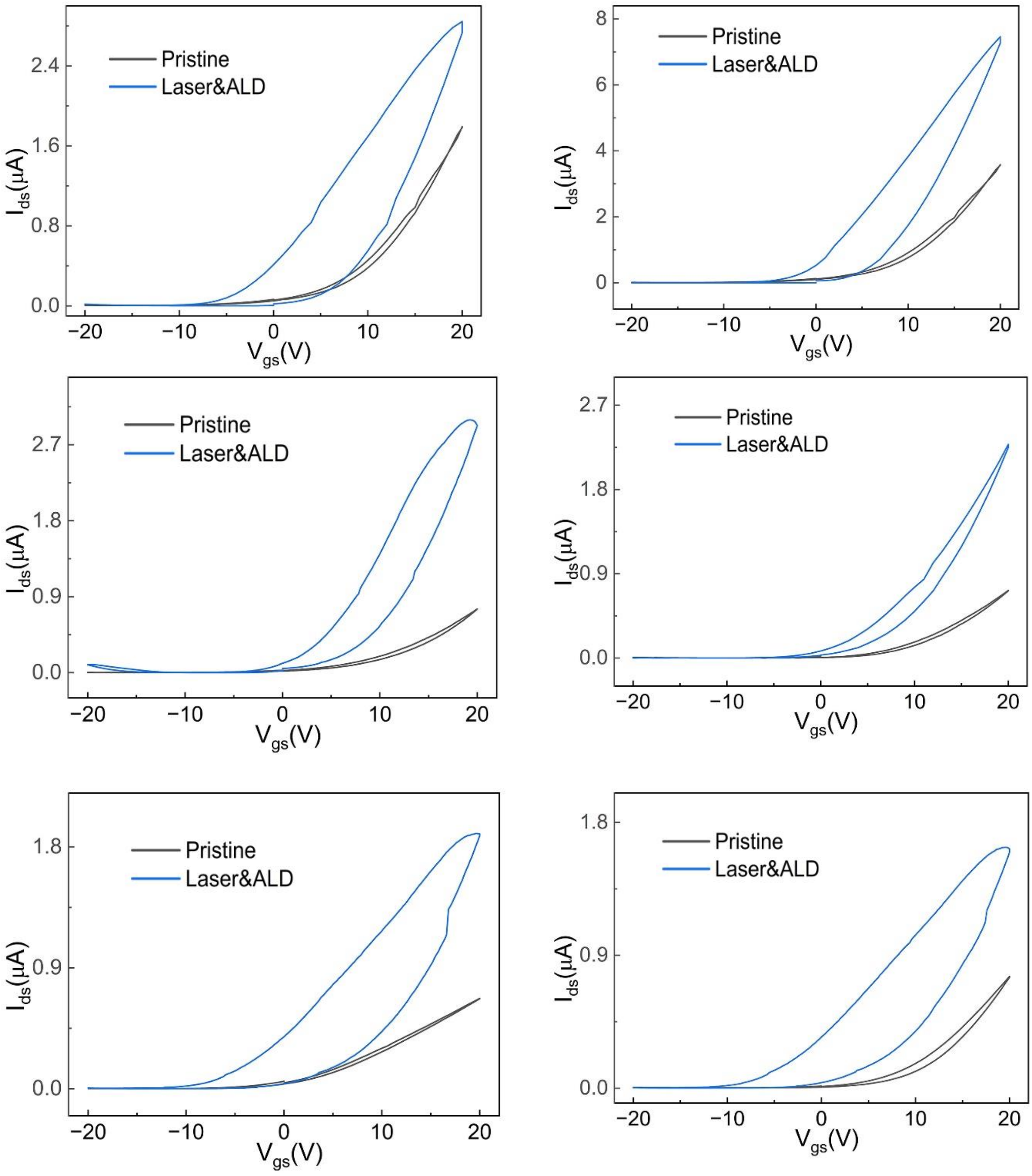

Figure S14. Electrical I_{ds}-V_{gs} characteristics of the pristine and processed (laser and ALD) $MoTe_2$ memtransistors for different devices.

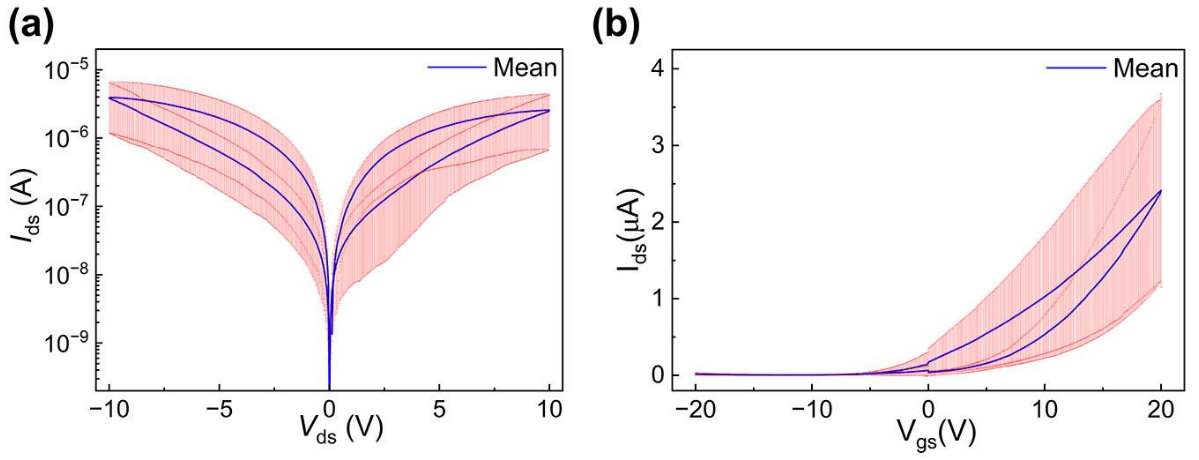

Figure S15. Evaluation of the device-to-device variation in processed $MoTe_2$ including mean and mean absolute deviation for a) memristor and b) memtransistor.

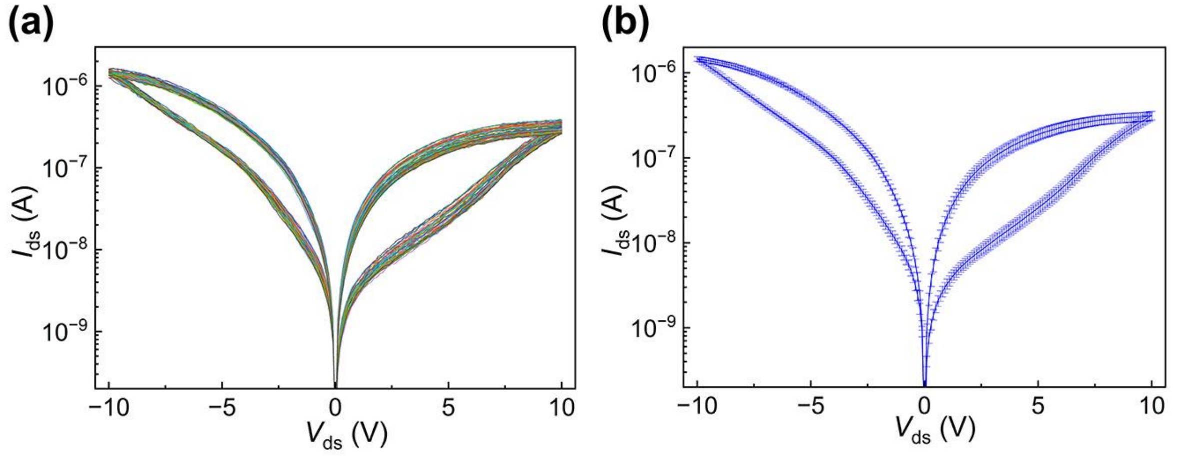

Figure S16. Evaluation of the cycle-to-cycle variation in processed $MoTe_2$ memristor over a) 50 consecutive I_{ds}-V_{ds} cycles and b) respective mean and mean absolute deviation.

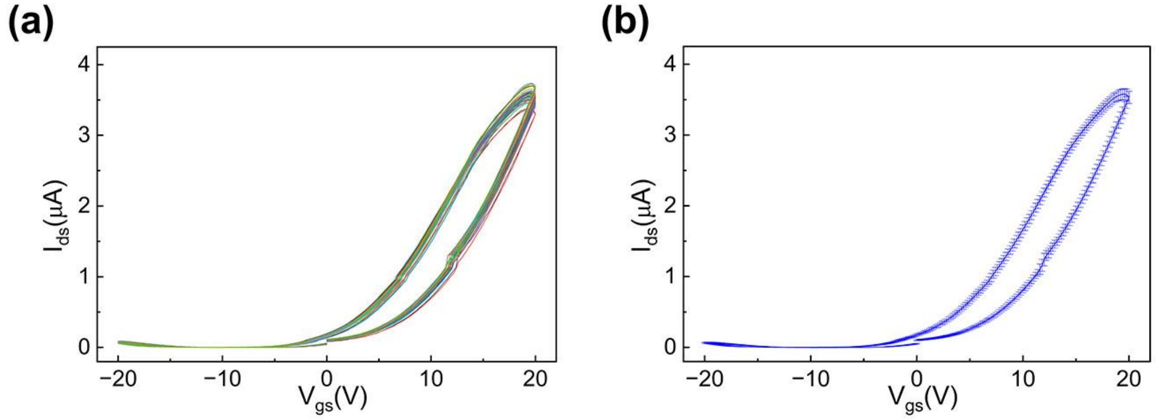

Figure S17. Evaluation of the cycle-to-cycle variation in processed $MoTe_2$ memtransistor over a) 50 consecutive I_{ds}-V_{gs} cycles and b) respective mean and mean absolute deviation.

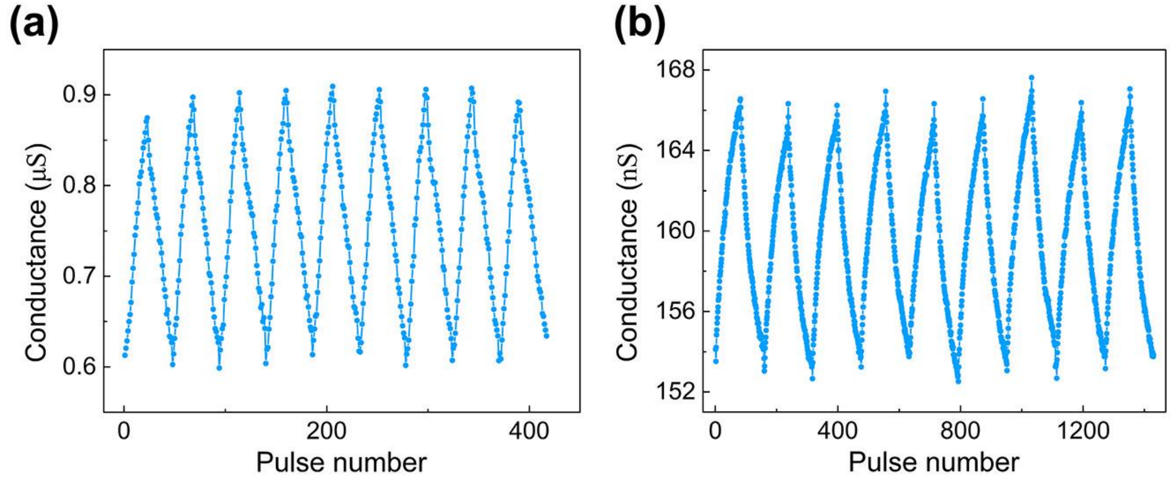

Figure S18. Repeatable potentiation and depression behaviors. a) Memristor device under ±10 V learning pulses with an 100 ms pulse width and +1 V reading pulses with a 100 µs pulse width. b) Memtransistor device under ±10 V pulses with a 60 ms pulse width.

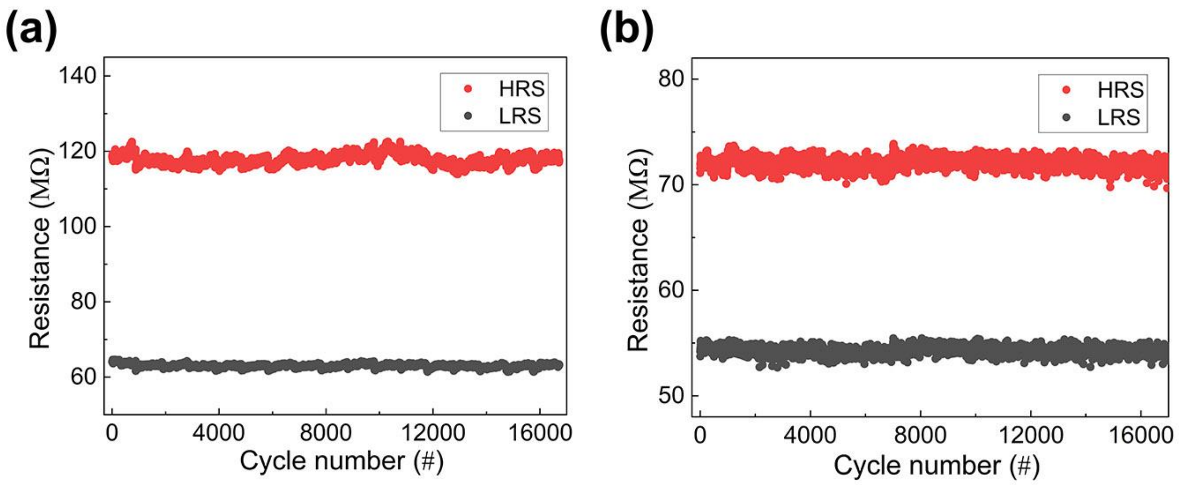

Figure S19. Endurance measurement of the processed $MoTe_2$ a) memristor and b) memtransistor.

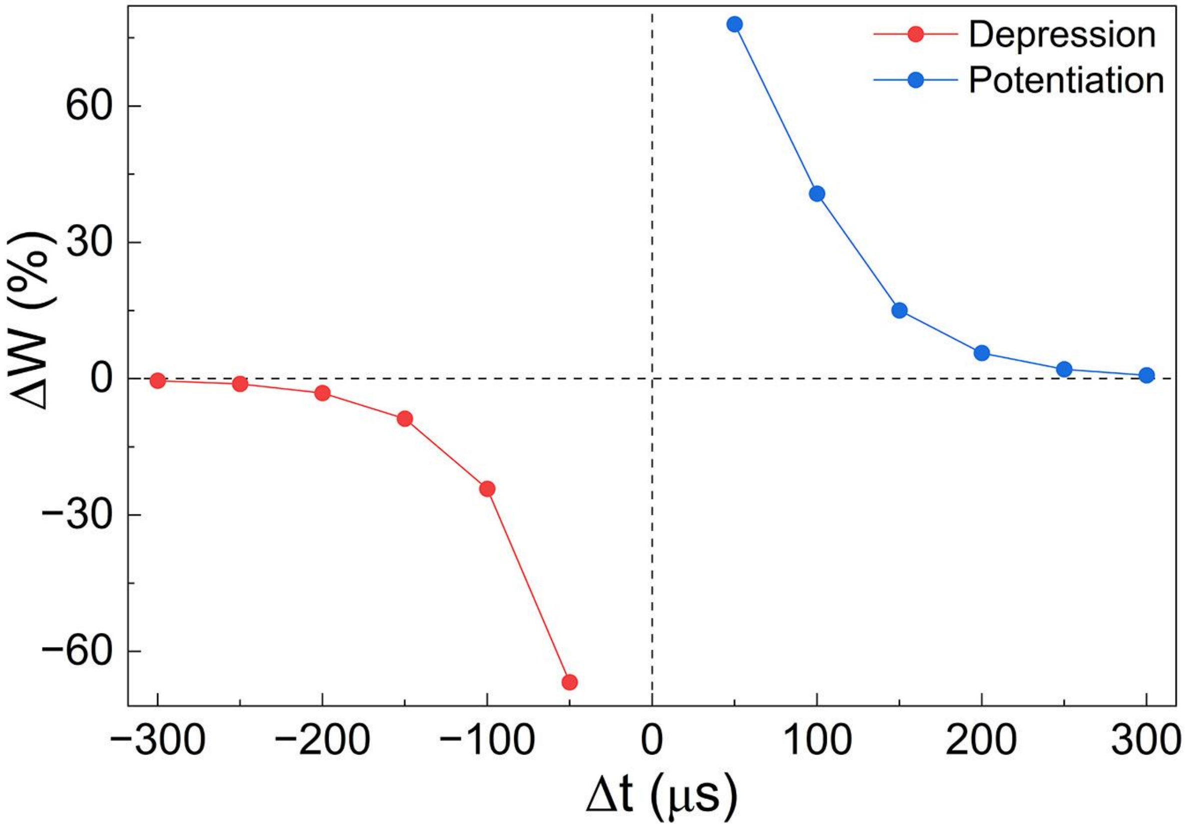

Figure S20. STDP behavior of processed $MoTe_2$ memristor
under two +10 V pulses with a 1 ms width.

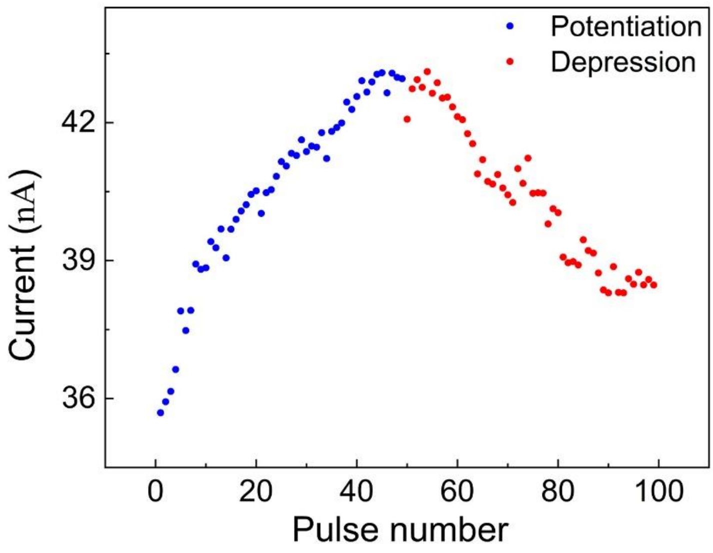

Figure S21. Analog switching behavior of memristor under ±3 V pulses with a 100 µs pulse width for Energy measurements.

Table S1. Comparison of the performance between this work and other reported 2D memristors.

2D Material	Treatment Method	DR Enhancement Ratio	Non-Linearity LTP/LTD	Symmetry %	Energy (pJ)	Endurance	Pulse Amplitude	Recognition %	Ref.
WS_2	Atomic Nb-doping	100	–	–	–	–	±30V	92.3	[1]
MoS_2	Argon plasma	100	2.81/2.79	90	30 fJ	100	±20V	97	[2]
MoS_2	Oxygen plasma	78	0.05/0.21	91	25	10^5	± (0.2 to 4) V	90	[3]
MoS_2	Argon plasma	20	2.1	–	–	–	+10V/-15V	94.3	[4]
MoS_2	UV-Ozone	10	–	–	–	–	–	–	[5]
$MoTe_2$	Mild oxidation	11	1.01/4.95	65	–	–	±10V	96	[6]
$MoTe_2$	Lithium-Ion Intercalation	–	0.55/0.21	89	3.2	100	±5V	90	[7]
$MoTe_2$	Laser/ALD	70	1.13/1.6	91	12	1.6×10^4	±10V	94	This work

4. Transfer Length Method Analysis for Pristine and Processed Devices.

To evaluate the contact resistance (R_c), we fabricated a new set of devices with channel lengths of 3, 4, 5, and 6 μm and measured their electrical transport characteristics. The contact resistance is extracted using the transfer length method (TLM) for both the pristine and processed devices. According to the TLM model, the total resistance R_t is given by:

$$R_t = 2R_c + R_{sh}\left(\frac{L}{W}\right) \tag{3}$$

where R_{sh} is the sheet resistance, L and W are the channel length and width. In the pristine device, the TLM plot yielded an intercept of 3.8 MΩ, whereas after processing the intercept decreased to 0.6 MΩ (see Figure S22). Therefore, the extracted contact resistance is R_c=1.9 MΩ in the pristine device and R_c=0.3 MΩ after processing, indicating a substantial suppression of the contact barrier after processing.

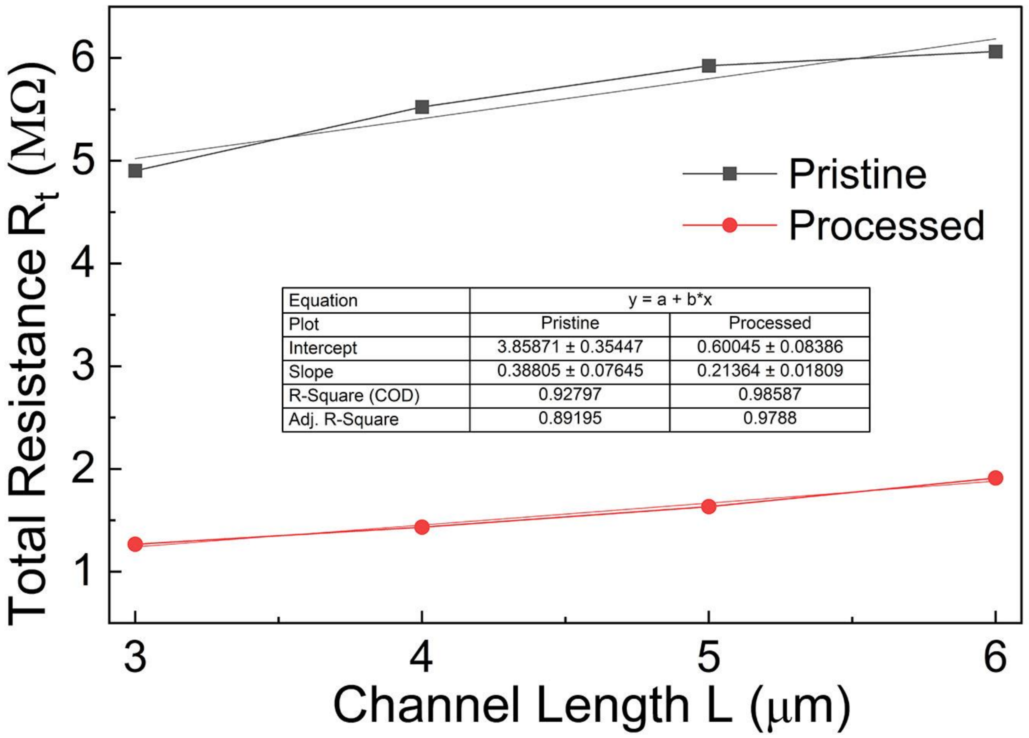

Figure S22. TLM measurements for devices a) pristine and b) processed (laser and ALD).

5. Trap Density Calculation for Pristine and Processed Devices.

To prove the increase of trap density after Al_2O_3 deposition, we calculated the density of interface states D_{it} for pristine and processed (laser and ALD) devices using below [8]

$$SS = \ln(10)\frac{KT}{q}\left(1 + \frac{qD_{it}}{C_{ox}}\right) \tag{1}$$

where SS is subthreshold slope $dV_g/d(logI_d)$, k is Boltzmann's constant, T is temperature, q is elementary charge, and C_{ox} is the oxide capacitance per unit area. Rearranging to solve for D_{it}:

$$D_{it} = \frac{C_{ox}}{q}\left(\frac{SS\ \times q}{\ln(10)\,KT} - 1\right) \tag{2}$$

Figure S23 shows that SS increased from 100 mV/dec for the pristine device to 150 mV/dec after processing. Correspondingly, the interface state density D_{it} increased from 5.2×10^{10} to $1.2 \times 10^{11}\ cm^{-2}\ eV^{-1}$, confirming the formation of additional trap states induced by Al_2O_3 deposition combined with laser treatment.

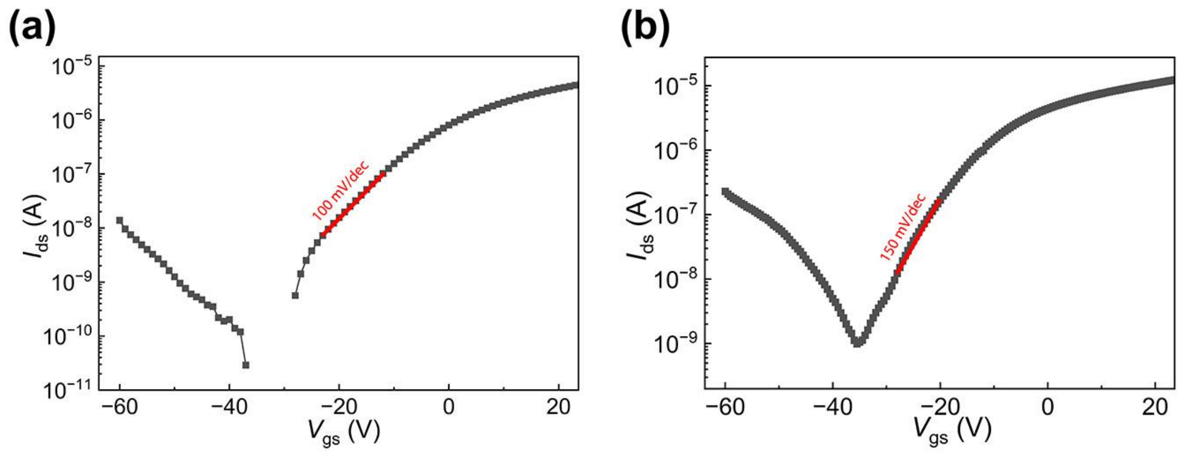

Figure S23. Gate transfer characteristics of memtransistor a) pristine and b) processed (laser and ALD).

6. Conduction Mechanism Analysis for Processed Memristor.

To understand the conduction mechanism in the processed $MoTe_2$ memristor, double-logarithmic I-V curves are plotted for forward and reverse bias regions, as shown in Figure S24. At lower voltages, the slope of the fitted curves is approximately 1.26 (forward bias) and 1.5 (reverse bias), indicating Ohmic conduction. As the voltage increases, the current deviates from linearity (slope ≈ 2.4), suggesting a transition to space-charge-limited conduction (SCLC). At higher voltages, the slope increases significantly to approximately 5 (forward bias) and 4.1 (reverse bias), suggesting a transition to trap-filled-limited (TLF) transport [9].

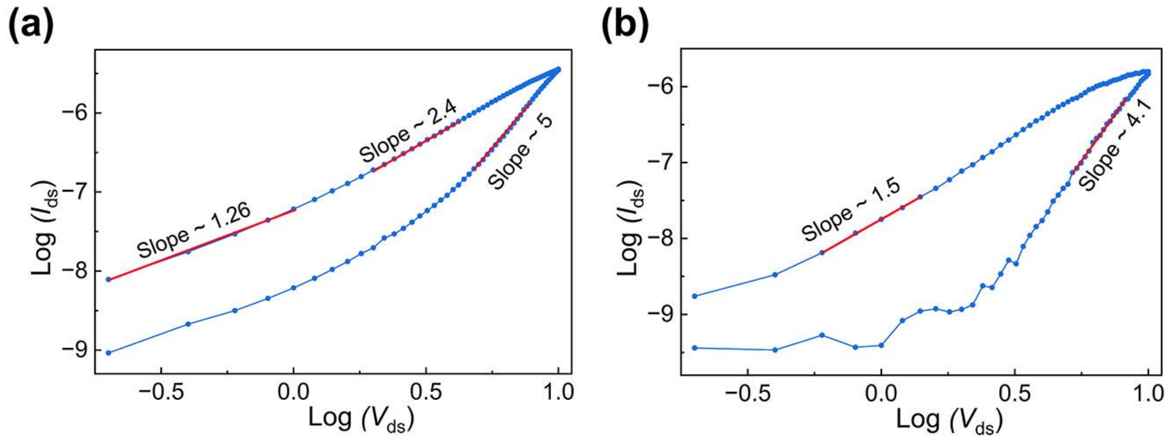

Figure S24. Double-logarithmic *I-V* plots for the processed $MoTe_2$ memristor.
a) forward bias (V > 0) and b) reverse bias (V < 0).

[1] K. Guan *et al.*, "Atomic Nb-doping of WS2 for high-performance synaptic transistors in neuromorphic computing," *Microsystems & Nanoengineering,* vol. 10, no. 1, p. 132, 2024/09/26 2024, doi: 10.1038/s41378-024-00779-1.

[2] M. Rajput *et al.*, "Defect-engineered monolayer MoS2 with enhanced memristive and synaptic functionality for neuromorphic computing," *Communications Materials,* vol. 5, no. 1, p. 190, 2024/09/16 2024, doi: 10.1038/s43246-024-00632-y.

[3] K. Varshney, P. Shukla, B. Prakash, D. M. Das, and B. Rawat, "Enhancing Resistive Switching Characteristics of MoS_2-Based Memristor Through O_2 Plasma Irradiation-Induced Defects," *IEEE Journal of the Electron Devices Society,* vol. 13, pp. 737–744, 2025, doi: 10.1109/JEDS.2024.3480356.

[4] D. Li, B. Ryu, J. Yoon, Z. Li, and X. Liang, "Improvement of analogue switching characteristics of MoS2 memristors through plasma treatment," *Journal of Physics D: Applied Physics,* vol. 53, no. 13, p. 135305, 2020/01/21 2020, doi: 10.1088/1361-6463/ab6572.

[5] B. Shin *et al.*, "Half-Oxidized MoS2-Based Memristor by UV-Ozone Treatment," *ACS Applied Electronic Materials,* vol. 7, no. 9, pp. 3737–3743, 2025/05/13 2025, doi: 10.1021/acsaelm.5c00031.

[6] B. Zhao *et al.*, "High-Performance 2D Ambipolar MoTe2 Lateral Memristors by Mild Oxidation," *Small,* vol. 20, no. 43, p. 2402727, 2024, doi: https://doi.org/10.1002/smll.202402727.

[7] R. H. Rupom *et al.*, "Ion-Induced Phase Changes in 2D MoTe2 Films for Neuromorphic Synaptic Device Applications," *ACS Nano,* vol. 19, no. 2, pp. 2529–2539, 2025/01/21 2025, doi: 10.1021/acsnano.4c13915.

[8] F. Ali *et al.*, "Achieving Near-Ideal Subthreshold Swing in P-Type WSe2 Field-Effect Transistors," *Advanced Electronic Materials,* vol. 10, no. 9, p. 2400071, 2024, doi: https://doi.org/10.1002/aelm.202400071.

[9] W. Huh *et al.*, "Heterosynaptic MoS2 Memtransistors Emulating Biological Neuromodulation for Energy-Efficient Neuromorphic Electronics," *Advanced Materials,* vol. 35, no. 24, p. 2211525, 2023, doi: https://doi.org/10.1002/adma.202211525.
